# A Pure Rotational Spectroscopic Study of Two Nearly-Equivalent Structures of Hexafluoroacetone Imine, (CF_3_)C=NH

**DOI:** 10.3390/molecules30092051

**Published:** 2025-05-05

**Authors:** Daniel A. Obenchain, Beppo Hartwig, Daniel J. Frohman, G. S. Grubbs, B. E. Long, Wallace C. Pringle, Stewart E. Novick, S. A. Cooke

**Affiliations:** 1 Institute of Physical Chemistry, University of Göttingen, Tammannstrasse 6, 37077 Göttingen, Germany; bhartwi@gwdg.de; 2 Department of Chemistry, Wesleyan University, 52 Lawn Avenue, Middletown, CT 06459, USA; dfrohman@wesleyan.edu (D.J.F.); wpringle@wesleyan.edu (W.C.P.);; 3 Department of Chemistry, Missouri University of Science and Technology, 104 Schrenk Hall, 400 W. 11th St, Rolla, MO 65409, USA; grubbsg@mst.edu; 4 Department of Chemistry and Biochemistry, James Madison University, Harrisonburg, VA 22807, USA; long2be@jmu.edu; 5 School of Natural and Social Sciences, Purchase College SUNY, 735 Anderson Hill Rd, Purchase, NY 10577, USA

**Keywords:** hexafluoroacetone imine, 1,1,1,3,3,3-hexafluoro-2-propanimine, rotational spectroscopy, large amplitude motions

## Abstract

Rotational spectra for hexafluoroacetone imine, the singly substituted ^13^C isotopologues, and the ^15^N isotopologue, have been recorded using both cavity and chirped pulse Fourier transform microwave spectrometers. The spectra observed present as being doubled with separations between each pair of transitions being on the order of a few tens of kilohertz which is consistent with a large amplitude motion producing two torsional substates. The observed splitting is most likely due to the combined motions of the CF3 groups, for which the calculated barrier is small. However, no transitions between states could be observed and, similarly, no Coriolis coupling parameters were required to achieve a satisfactory fit for the transition frequencies. Hence, and somewhat curiously, the two states have been fit independently of each other such that the two states may simply be considered near-equivalent conformers. The structural properties of hexafluoroacetone imine are compared with two isoelectronic molecules hexafluoroisobutene and hexafluoroacetone. Rotational constants, quartic centrifugal distortion constants, and the ^14^N nuclear electric quadrupole coupling tensor have been determined and are presented together with supporting quantum chemical calculations.

## 1. Introduction

**Figure 1:**
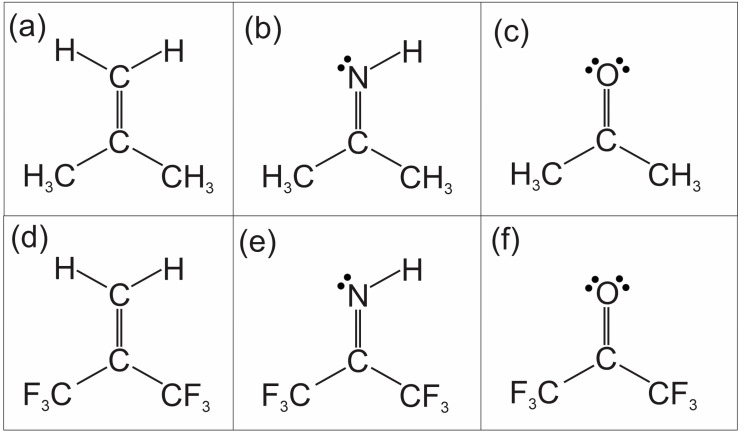
Two series of isoelectronic molecules. (**a**–**c**) is isobutene, acetone imine, and acetone. (**d**–**f**) are the hexafluorinated analogs.

Hexafluoroacetone imine may be considered a member of a grouping of six molecules shown in Figure 1. This grouping consists of two isoelectronic series, the first being isobutene, acetone imine, and lastly acetone, and the second being the hexafluorinated analogs of the first series. Except for hexafluoroacetone imine, all these molecules have had their rotational spectra recorded at high resolution. These spectra are reported, or most recently discussed, in the following references [1,2,3,4,5]. Isobutene and acetone are well-known examples of rotational spectra in which the effects of the internal rotation of two equivalent methyl tops are manifested. At high resolution, rotational transitions from these types of molecules will appear as quartets owing to transitions occurring from within the *AA*, *EE*, *AE*, and *EA* torsional sub-levels.

These spectra have been interpreted to yield effective CH_3_ internal rotation tunneling barriers of 761(1) cm^−1^ for isobutene [1] and 251.4(26) cm^−1^ for acetone [3]. One would correctly assume that the tunneling barrier to CH_3_ internal rotation in acetone imine would be intermediate between isobutene and acetone. However, the spectra are more complex owing to the two CH_3_ rotors being non-equivalent. In that case, the effects of the two non-equivalent CH_3_ rotors result in five torsional substrates, one with *A*_1_ symmetry and four doubly degenerate states with *E* symmetry. Hence, rotational transitions from within these substates present to the observer as quintets. An analysis of these spectra by Zou et al. [2] has resulted in internal rotation barrier heights of 531.956(64) cm^−1^, attributed to the CH_3_ furthest from the imine hydrogen, and 465.013(26) cm^−1^, attributed to the CH_3_ nearest to the imine hydrogen. Taken as a series, then we observe that the CH_3_ barriers to internal rotation increase in the order of increasing hydrogenation of the apex atom, i.e., in the order O, N-H, H-C-H. From a purely classical mechanics standpoint, this is rational in the sense that the apex hydrogen(s) clearly impede the CH_3_ internal rotations.

We then consider the hexafluorinated analogs. The rotational spectrum of hexafuoroacetone has been recorded at high resolution with spectral line widths on the order of 7 kHz at full width, half maximum height [5]. However, none of the transitions observed for hexafluoroacetone were observed as multiplets, or broadened as they were for acetone. Now, it is generally true that, the higher the barrier heights to internal motion the closer together in energy will be the torsional substates and, hence, the rotational transitions from within those different torsional substates will appear closer together in frequency. Given that the barrier heights to CF_3_ internal rotation are significantly higher than those for CH_3_ internal rotation, together with the very small internal rotational constant for a CF_3_ group, *F* ≈ 10 GHz (compared to that for a CH_3_ group, *F* ≈ 160 GHz), then effects due to CF_3_ internal rotation are most often unresolvable. A helpful discussion on this topic is presented in reference [6]. Accordingly, the rotational spectrum of hexafluoroacetone presents like that of an “ordinary” semirigid rotor, with no evidence of any internal motions.

Given that isobutene has a higher barrier to CH_3_ internal rotation compared to that of acetone, it may be assumed that the barrier to CF_3_ internal rotation in hexaflouroisobutene would be higher than that in hexafluoroacetone and, therefore, would have a rotational spectrum with no observable evidence of internal motion. However, the recorded rotational spectrum of hexafluoroisobutene presented as being doubled with spacings between the rotational transitions being on the order of tens of megahertz. The spectral analyses revealed that the bis-trifluoromethyl groups of hexafluoroisobutene are staggered in the equilibrium configuration, and that a novel, out-of-phase rotation through an F-CCC-F planar configuration with a low barrier (<100 cm^−1^), leads to the observed doubled rotational spectra [4]. This bistrifluoromethy effect has subsequently been explored in related molecules [7,8].

In this article, we present the measurement and analyses of rotational spectra for the remaining member of the molecules in Figure 1, hexafluoroacetone imine. We ask the question of how well the spectra are present, like that of hexafluoroacetone or that of hexafluoroisobutene. And further, how do we interpret the spectra?

## 2. Results

### 2.1. Spectral Analysis

**Figure 2:**
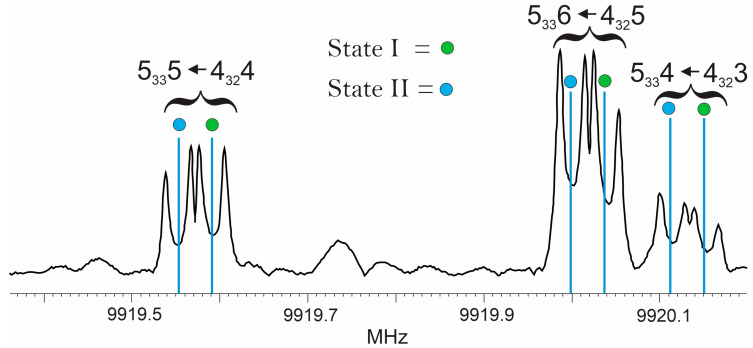
A section of the spectra recorded for hexafluoroacetone imine using a cavity Fourier transform microwave spectrometer. The quantum number assignments given follow *J′_K−1K+1_F* ← *J″_K−1K+1_F*. The portion of spectra shown is the Fourier transform of 75 free-induction decays averaged together. Transitions appear as Doppler doublets and the average of the two Doppler components is taken as the line center. The vertical lines indicate the predicted frequencies for these transitions using the rotational constants given in Table 1.

The ground-state rotational spectrum of hexafluoroacetone imine, and all of the singly substituted ^13^C and ^15^N isotopologues, exhibits both a-type and b-type transitions. The spectra consist of “doubled” transitions separated by a few tens of kilohertz, we refer to a transition as either belonging to State I or State II. An example set of transitions is shown in Figure 2.

For both State I and State II, transition quantum number assignments and measured frequencies are available in the Supplementary Data. For each state, spectroscopic constants were fitted to the observed frequencies using the SPCAT/SPFIT software [9]. The Hamiltonian used was of the simple form H = H_R_ + H_Q_ constructed in the coupled symmetric rotor basis set **I** + **J** = **F**. The Watson A reduction in the I^r^ representation was chosen for the semirigid rotor Hamiltonian H_R_ [10], but only terms up to the fourth power in angular momentum were required. The second operator, H_Q_, represents the well-known interaction energy of the nuclear electric quadrupole moment with the electric field gradient at the nitrogen nucleus. The term H_Q_ is not needed for the ^15^N isotopologue as the nuclear spin, I = 1/2, and it therefore does not possess a quadrupole moment. In all cases, the χ_aa_ and χ_cc_ were used in the fits with χ_bb_ being determined from the requirement that χ_aa_ + χ_bb_ + χ_cc_ = 0. The determined spectroscopic constants for the two states of the parent isotopologue are presented in Table 1. The spectroscopic constants of the minor isotopologues are presented in Table 2 (State I), and Table 3 (State II). Many attempts were made to (i) locate transitions that span States I and II, and (ii) to use Coriolis constants in order to link the states. However, only those constants shown in Table 1, Table 2 and Table 3 were required to fit the transition data sets.

### 2.2. Theoretical Calculations

**Figure 3:**
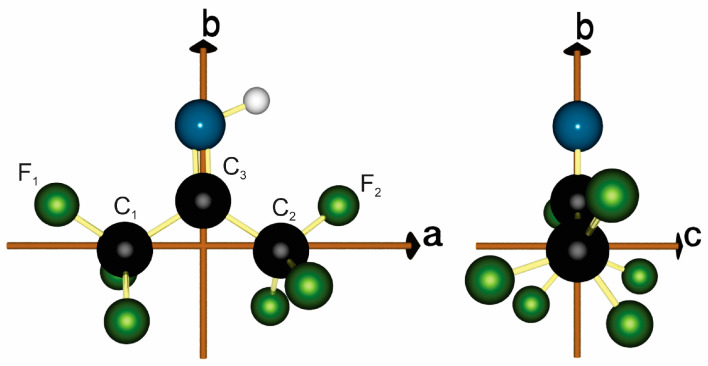
Hexafluoroacetone imine calculated at the MP2/6-311G++(2d,2p) level in the *ab-* and *bc-* planes of its principal axes system.

The MP2/6-311G++(2d,2p) [11,12,13,14] equilibrium geometry of hexafluoroisobutene is shown in Figure 3 and this structure produces the rotational constants shown in Table 1.

Calculated centrifugal distortion constants are also shown in Table 1. It is easily seen that the agreement is very good, with only small discrepancies appearing for some of the centrifugal distortion constants. This is unsurprising given the likely internal motions.

**Figure 4:**
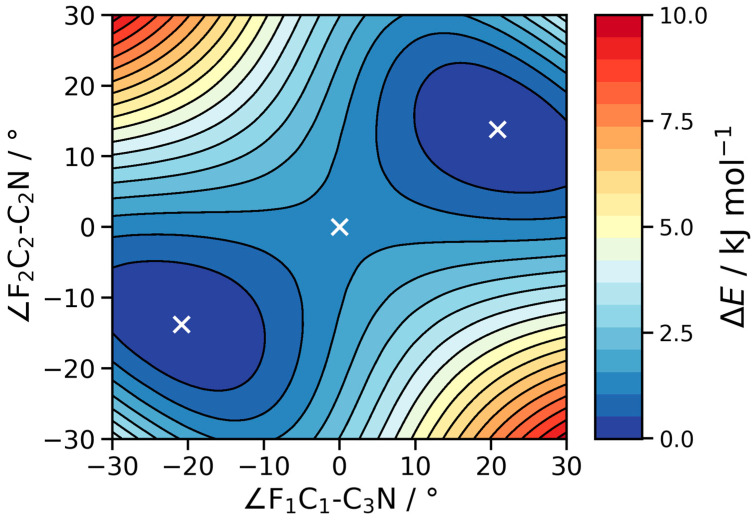
A 2-D potential energy scan for hexafluoroacetone imine. Stationary points are shown with a white cross. Please refer to Figure 3 for the atomic number scheme, and to the text for further discussion.

As discussed in the introduction, curiosity surrounds the possibility of observing the hexafluoroacetone imine spectra effects due to a similar internal motion to that observed for hexafluoroisobutene. The relaxed 2D potential energy scan for hexafluoroisobutene is shown in Figure 4 (see Figure 3 for atom labeling). For reference, the staggered nature of the F-CCC-F atoms in the equilibrium structure is shown on the right-hand side of Figure 3.

The zero-degree/zero-degree center of the scan corresponds to a planar F-CCC-F configuration. A one-dimensional, diagonal slice (lower left minimum through to upper right minimum) of the potential energy scan for hexafluoroacetone imine shows a double minimum with a barrier height of approximately 116 cm^−1^. The barrier heights at other levels of theory are shown in Table 4, and also in the Supplementary Tables, and are generally consistent. This is, approximately, double that of the analogous barrier height in hexafluoroisobutene, and using alternative quantum mechanical methods Shahi and Arunan [8] have shown the barrier quadruples in magnitude. For reference, the Shahi and Arunan calculations have been reproduced and are shown in the Supplementary Data. The increased barrier height for this out-of-phase rotation of CF_3_ groups through a planar F-CCC-F local maximum is consistent with the rotational transitions within each torsional substrate being closer together in frequency than for those observed in hexafluoroisobutene.

The results of a nudged-elastic-band scan are shown in the Supplementary Data for hexafluoroacetone, hexafluoroacetone imine, and hexafluoroisobutene. The angles used here are the average of the two dihedral angles defining the rotation of the CF3 groups. The scans illustrate that both the width as well as the barrier height significantly increase in going from hexafluoroisobutene to hexafluoroacetone imine to hexafluoroacetone. From the scans, you can also see that in hexafluoroacetone and hexafluoroacetone imine the lone pair side dihedral angles are actually very similar.

For the purpose of deriving semi-experimental equilibrium structures r_e_^SE^, we conducted vibrational perturbation theory of second-order calculations (VPT2) [15] to obtain vibrational corrections for the rotational constants. In particular, we used Gaussian’s generalized VPT2 (GVPT2) implementation [16,17]. Note that the VPT2 calculation was conducted separately from the geometry optimization.

### 2.3. Structure

From the calculations detailed above, a very accurate structure was determined that reproduced the observed rotational constants very well. The best method used was the PBE0-D3(BJ)/aug-cc-pVTZ level of the theory and key structural features are presented in Table 5. The computed rotational constants are shown in Table 6.

Attempts were made to perform r_0_, r_e_^SE^ (semi-experimental [18]) and r_m_^(2)^ [19] fits using the determined experimental rotational constants and, where appropriate, with quantum mechanically calculated vibrational parameters. For a comprehensive review of these approaches, please see reference [20]. For the C=N bond, the values determined were satisfactory, but in all the attempts, the two unique C-C bonds are not in agreement with the calculated structure, having large uncertainties in the bonds, and non-converging fits. The r_m_^2^ structure converges with both c_a_ and d_a_, but again the bonds have non-physical values. It is most likely that these failures have to do with the effective nature of the rotational constants used together with, possibly, poorly captured large amplitude motions in the quantum mechanical calculations.

Given the availability of the rotational constants for all the singly substituted ^13^C- and ^15^N-isotopologues Kraitchman analysis [21] we can obtain the substitution principal atomic coordinates of each of the substituted atoms, with the origin of these coordinates being the center of mass of the parent isotopologue. The results are shown in Table 7. As expected, it is found that both State I and II share nearly-equivalent structures which agree well with the quantum mechanical calculations.

Regarding the structure, it is interesting to compare the second moment in the direction of the *c*-principal axes, *P_cc_* = $\sum_{i}m_{i}c_{i}^{2}$, for the three molecules: hexafluoroisobutene, hexafluoroacetone imine, and hexafluoroacetone. All these structures have two CF_3_- groups which are staggered with respect to each other, and so the only contributions to *P_cc_* will be from the six out-of-plane fluorine atoms. The *P_cc_* values may be determined from the experimental rotational constants and are found to be 89.20 amu Å^2^, 88.79 amu Å^2^, and 89.15 amu Å^2^, respectively. For two CF_3_ groups, Bohn [22] has shown that the anticipated value of *P_cc_* should be approximately 90 amu-Å^2^. This is in good agreement with the present data set and is indicative of the staggered/helical tendencies of many perfluorinated molecules [23].

### 2.4. Nitrogen Nuclear Electric Quadrupole Coupling Tensor

The ^14^N-nitrogen nuclear quadrupole coupling tensor components for hexafluoroacetone imine are compared to those for CH_2_NH [24] and CF_2_NH [25,26] in Table 8. Direct comparison is difficult as each molecule has its own principal axes system. However, in all three cases, the *c*-principal axes are perpendicular to the C=N-H plane and therefore the χ_cc_ values are comparable. For χ_cc_, we find that the magnitudes change in the order CH_2_NH > (CF_3_)_2_CNH > CF_2_NH. This trend may be rationalized by an appeal to the electronegativities of H, F, and CF3, and it is found that the ordering of electronegativities on the Allred-Pauling scale [27,28] is F (3.98) > CF3 (2.99) > H (2.20), the opposite trend to that observed for χ_cc_. Nuclear quadrupole coupling tensors are often related to ionicity [29] where the quadrupole coupling tensor for an ion will be very close to zero owing to the spherical symmetry of either empty (or full) *p*-orbitals. So, one may rationalize that χ*_cc_* will decrease in magnitude with an increase in the electronegativity of the attached groups which is consistent with experimental observations.

## 3. Discussion

**Figure 5:**
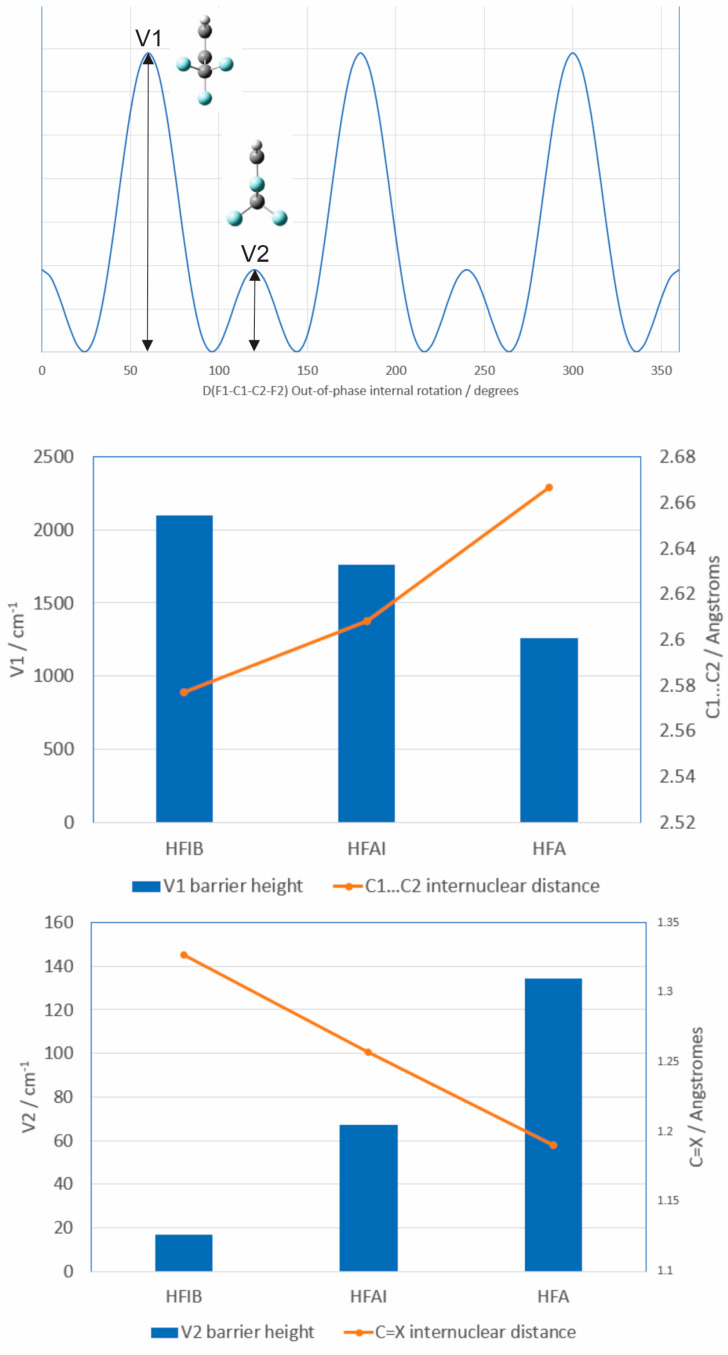
Top: an example, i.e., not to any scale but just showing form, of a relevant potential energy plot with the x-axis being the F1-C1-C2-F2 dihedral angle. Middle: Bar chart showing how, for the series HFIB, HFAI, HFA the barrier V1 decreases as the distance between the C1 and C2 atoms increases. Bottom: Bar chart showing how the V2 barrier height increases as the C=X distance decreases, X=C, N, and O. HFIB is hexafluoroisobutene, HFAI is hexafluoroacetone imine, and HFA is hexafluoroacetone. See Figure 3 for the atom labeling scheme.

The pure rotational spectrum of hexafluoroacetone imine presents as consisting of two nearly-equivalent conformers. However, it is undoubtedly the case that these two sets of measured rotational transitions arise from two torsional substates, State I and State II, resulting from a relatively low barrier between two equivalent F-CCC-F staggered configurations. The torsional substates most likely arise from an out-of-phase internal rotation of the two CF_3_ groups which is governed by an unusual potential energy function with six minima, three high barriers (V1) and three low barriers (V2) as observed in the related molecule hexafluoroisobutene [4]. The substrates arise via tunneling through the low barrier. An example of the potential energy function is given in Figure 5. For the hexafluorinated species, the global minimum corresponds to a staggered F_1_-CCC-F_2_ configuration exemplified in Figure 3.

Careful calculations by Shahi and Arunan [8] have revealed that for the isoelectronic sequence of molecules hexafluoroisobutene (HFIB), hexafluoroacetone imine (HFAI), and hexafluoacetone (HFA) the barrier height V1 varies as HFIB > HFAI > HFA, whereas the V2 barrier height varies as HFA > HFAI > HFIB. In all three cases, the V1 barrier height is too high for tunneling effects to manifest in the observed spectra consistent with the absence of groupings of rotational transitions from within four or five torsional substates. The V2 tunneling barrier height is more nuanced. With the molecule unable to tunnel through V1, we are left with a tunneling motion through a much lower barrier separating two equivalent F-CCC-F staggered configurations, more akin to a double minimum potential often observed in ring-puckering problems. The barrier V2 is lowest for hexafluoroisobutene consistent with rotational transitions from the two torsional states being separated by tens of MHz. Whereas the barrier V2 is highest for hexafluoroacetone consistent with no observed “doublets” in the rotational spectrum. Hexafluoroacetone imine is intermediate, again consistent with the observation of paired rotational transitions separated by tens of kHz.

The above trends in both V1 and V2 may be rationalized through an appeal to the molecular geometries. In regards to the trend in V1, we note that all three molecules possess a C_1_-C_3_-C_2_ structural component, see Figure 3. It is found that the distance C_1_…C_2_ decreases in the order HFIB > HFAI > HFA. A large value of C_1_…C_2_ means that the two CF_3_-groups are further away from one another, and thus easier to rotate, compared to a small value of C_1_…C_2_ which corresponds to the CF_3_-groups being closer together. The correspondence between V1 and C_1_…C_2_ is shown in Figure 5. In regards to V2 in which the top of the smaller barrier corresponds to a planar F_1_-CCC-F_2_ (see Figure 5), it is useful to compare the C=X distance where X = CH_2_, NH, and O. This C=X distance decreases in the order HFIB > HFAI > HFA. The larger C=X distance in hexafluoroisobutene corresponds to a small V2, i.e., it is easier for F-CCC-F to pass through a planar configuration, compared to the longer C=X distance in hexafluoroacetone where the planar F-CCC-F configuration is more greatly interfered with by the oxygen lone pairs. The correspondence between V2 and C=X is also shown in Figure 5.

**Figure 6:**
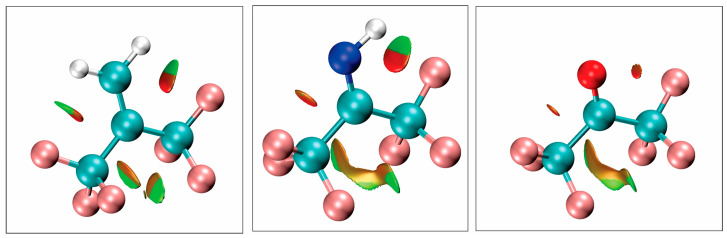
From left to right regions of most attractive (green) and most repulsive (red) interactions for hexafluoroisobutene, hexafluoroacetone imine, and hexafluoroacetone. Colors between red and green indicate areas of intermediate attraction/repulsion.

The above arguments are supported and augmented by calculations that reveal non-covalent interactions. Following the work of Johnson et al. [30] non-covalent interactions based on the electron density and its derivatives have been located for hexafluoroisobutene, hexafluoroacetone imine, and hexafluoroacetone. The results are presented in Figure 6. In this figure, green indicates weak attractive interactions whereas the orange and reddish colors indicate repulsive interactions. To obtain these plots, the electron density at the B3LYP level was analyzed using the Multiwfn [31,32] program and then visualized using VMD [33].

Within the figure, it is observed that in going from hexafluoroacetone-to-hexafluoroacetone imine to hexafluorisobutene the attractive interactions increase between the CF_3_ groups and O, NH and CH_2_, which in turn explains why the rotation of the CF_3_ groups becomes easier in that series. It is also observed that interactions with the lone pairs are purely repulsive whereas interactions with the NH and CH_2_ are partially attractive.

## 4. Materials and Methods

### 4.1. Experimental Methods

Hexafluoroacetone imine (95%, b.p. 16.5°) was purchased from Synquest Labs and used without further purification. A gas tank was pressurized to approx. 0.25 bar of hexafluoroacetone imine and then diluted with argon to a final pressure of approximately 6 bar. This solution of gases was pulsed through a solenoid valve into vacuum chambers held at approximately 10^−4^ bar causing a rotational cooling to approximately T^rot^ ≈ 3 K. Rotational spectra were recorded between 5.5 GHz and 21 GHz on two types of spectrometers first a chirp-pulsed Fourier transform spectrometer, and then a Balle-Flygare cavity Fourier transform spectrometer. Both spectrometers have been explained in detail elsewhere [34,35,36,37,38]. Common to both instruments was the use of microsecond pulses of microwave radiation to bring about bulk rotational coherence of the cold target molecules. A free-induction decay, FID, was collected as a function of time as the bulk coherence is lost. FIDs are averaged to reduce random noise, and were then fast Fourier transformed to produce a frequency domain spectrum. The key difference between the spectrometers is that the chirp-pulsed spectrometer utilizes a microwave pulse of radiation containing a sweep, i.e., chirp, of frequencies from, say, 8–18 GHz. This chirp was then greatly amplified to powers of, in the present case, 5 W and then broadcast onto the molecules through a horn antenna. The FIDs collected were amplified and consisted of 800,000 points which was fast Fourier transformed on a suitably broad-banded oscilloscope. Line widths with this method were typically on the order of 80 kHz and line centers possess uncertainties of approximately 10 kHz. In the cavity experiment, monochromatic pulses of microwave radiation are amplified using a Fabry-Perot resonator which resulted in very high interrogation path lengths. FIDs were collected in the same way as above; however, the FIDs consist of approximately 1600 data points and, after mixing down, were fast Fourier transformed on a narrow band PC oscilloscope card. Line widths with this method were approximately 10 kHz and line center uncertainties were approximately 2 kHz.

### 4.2. Quantum Chemical Calculations

Initially, geometry optimizations and vibrational frequencies were calculated at the MP2/6-311G++(2d,2p) [11,12,13,14] level of theory using the Gaussian 16 rev. B01 software [39]. This method was relatively fast and sufficient to allow initial assignments of experimental spectroscopic transitions.

Higher-level quantum mechanical calculations were also pursued. We utilized the commonly used B3LYP [40,41,42,43], PBE0 [44,45] and CAM-B3LYP [46] hybrid density functionals as well as the B2PLYP [47,48] and DSD-PBEP86 [49,50] double-hybrid functionals. The CAM-B3LYP and DSD-PBEP86 functionals may be more accurately referred to as a range-separated hybrid functional and spin component scaled double-hybrid functional, respectively. To compensate for the inaccurate description of dispersion interactions within density functionals, Grimme’s D3 dispersion correction was used throughout in conjunction with Becke-Johnson damping (D3(BJ)) [51,52,53]. As a basis set, we utilized Dunning’s augmented triple zeta basis set aug-cc-pVTZ [54,55].

Geometry optimizations were carried out using the *VeryTight* optimization criterion and the very dense *SuperFine* integration grid. For the transition state search, the Hessian matrix was recomputed exactly at every third step. Subsequently, analytic harmonic frequency calculations were conducted to confirm the presence of a minimum of (first order) transition states. In addition, we carried out 2D relaxed surface scans with the B3LYP, PBE0 and CAM-B3LYP functionals to better understand the potential energy surface. To this end, we varied the F_1_C_1_C_3_N and F_2_C_2_C_3_N dihedral angles from 30° to −30° in steps of 2° (see Figure 3 for atom labeling). To reduce computational cost during the scan, the optimization criterion was lowered to *Tight*.

The zero-degree/zero-degree center of the scan corresponds to a planar F-CCC-F configuration. A one-dimensional, diagonal slice (lower left minimum through to upper right minimum) of the potential energy scan for hexafluoroacetone imine shows a double minimum.

For deriving semi-experimental equilibrium structures r_e_^SE^, we conducted vibrational perturbation theory of second-order calculations (VPT2) [15] to obtain vibrational corrections for the rotational constants. In particular, we used Gaussian’s generalized VPT2 (GVPT2) implementation [16,17]. Note that the VPT2 calculation was conducted separately from the geometry optimization.

Lastly, Nudged-Elastic-Band [56] scans for the fluorinated variants using ORCA 6.0.1 [57,58] and 60 images at the B3LYP-D3(BJ)/aVTZ level were performed to further investigate the barrier heights.

## Figures and Tables

**Table 1:** Calculated and experimentally determined effective ground-state rotational constants, centrifugal distortion constants, and nitrogen nuclear electric quadrupole coupling constants for hexafluoroacetone imine.

	MP2/6-311G++(2d,2p)	State I	State II
*A* (MHz)	2158.610	2170.17085(35)	2170.16744(35)
*B* (MHz)	1045.446	1043.25444(14)	1043.24681(14)
*C* (MHz)	937.933	936.37030(13)	936.37059(13)
Δ*_J_* (kHz)	0.054	0.0576(10)	0.0525(10)
Δ*_JK_* (kHz)	0.15	0.2047(62)	0.2219(62)
Δ*_K_* (kHz)	−0.11	−0.363(26)	−0.457(26)
δ*_J_* (kHz)	0.0061	0.00734(44)	0.00471(43)
δ*_K_* (kHz)	−0.35	−0.475(41)	−0.563(41)
χ_aa_ (MHz)	-	−5.0732(33)	
χ_bb_ (MHz)	-	2.9924(40)	
χ_cc_ (MHz)	-	2.0809(40)	
RMS (kHz) ^1^	-	2.7	
*N*	-	176	177

^1^ Microwave root mean square = $\sqrt{\sum {[\left(obs-calc\right)}^{2}]/N}$ where *N* is the number of transitions.

**Table 2:** Experimentally determined effective ground-state rotational constants, centrifugal distortion constants, and nitrogen nuclear electric quadrupole coupling constants for the parent and isotopologues for State I of hexafluoroacetone imine.

	Parent	^13^C_1_	^13^C_2_	^13^C_3_	^15^N
*A* (MHz)	2170.17085(35)	2170.2055(79)	2170.1985(44)	2165.3028(24)	2134.1858(28)
*B* (MHz)	1043.25444(14)	1039.72015(93)	1039.65915(41)	1043.33695(20)	1043.25063(14)
*C* (MHz)	936.37030(13)	933.51356(21)	933.47028(12)	935.530070(95)	929.59788(10)
Δ*_J_* (kHz)	0.0576(10)	[0.0576]	[0.0576]	[0.0576]	[0.0576]
Δ*_JK_* (kHz)	0.2047(62)	[0.2047]	[0.2047]	[0.2047]	[0.2047]
Δ*_K_* (kHz)	−0.363(26)	[−0.363]	[−0.363]	[−0.363]	[−0.363]
δ*_J_* (kHz)	0.00734(44)	[0.00734]	[0.00734]	[0.00734]	[0.00734]
δ*_K_* (kHz)	−0.475(41)	[−0.475]	[−0.475]	[−0.475]	[−0.475]
χ_aa_ (MHz)	−5.0732(33)	−5.052(31)	−5.109(24)	−5.070(21)	-
χ_bb_ (MHz)	2.9924(55)	2.97(16)	2.974(89)	2.961(70)	-
χ_cc_ (MHz)	2.0809(40)	2.08(16)	2.135(86)	2.109(67)	-
RMS (kHz) ^1^	2.7	2.8	4.3	1.9	2.9
*N*	176	28	39	27	23

^1^ Microwave root mean square = $\sqrt{\sum {[\left(obs-calc\right)}^{2}]/N}$ where *N* is the number of transitions.

**Table 3:** Experimentally determined effective ground-state rotational constants, centrifugal distortion constants, and nitrogen nuclear electric quadrupole coupling constants for the parent and isotopologues for State II of hexafluoroacetone imine.

	Parent	^13^C_1_	^13^C_2_	^13^C_3_	^15^N
A (MHz)	2170.16744(35)	2170.2155(79)	2170.2607(41)	2165.2925(25)	2134.1836(21)
B (MHz)	1043.24681(14)	1039.71300(93)	1039.65821(44)	1043.32924(20)	1043.24306(13)
C (MHz)	936.37059(13)	933.51364(21)	933.46923(11)	935.530474(96)	929.59823(10)
Δ_J_ (kHz)	0.0525(10)	[0.0525]	[0.05764]	[0.05764]	[0.05764]
Δ_JK_ (kHz)	0.2219(62)	[0.2219]	[0.2046]	[0.2046]	[0.2046]
Δ_K_ (kHz)	−0.457(26)	[−0.457]	[−0.364]	[−0.364]	[−0.364]
δ_J_ (kHz)	0.00471(43)	[0.00471]	[0.00738]	[0.00738]	[0.00738]
δ_K_ (kHz)	−0.563(41)	[−0.563]	[−0.473]	[−0.473]	[−0.473]
χ_aa_ (MHz)	−5.0732(33)	−5.052(31)	−5.109(24)	−5.070(21)	-
χ_bb_ (MHz)	2.9924(55)	2.97(16)	2.974(89)	2.961(70)	-
χ_cc_ (MHz)	2.0809(40)	2.08(16)	2.135(86)	2.109(67)	-
RMS (kHz) ^1^	2.7	3.7	4.3	1.9	2.9
*N*	177	28	39	27	23

^1^ Microwave root mean square = $\sqrt{\sum {[\left(obs-calc\right)}^{2}]/N}$ where *N* is the number of transitions.

**Table 4:** Overview of the electronic (Δ*E*_el_^‡^) and zero-point corrected (Δ*E*_0_^‡^) barrier for the inversion. The singular imaginary mode ($\tilde{v}$_imag_) is also given associated with the transition state. All methods used (i) Grimme’s dispersion correction was used throughout in conjunction with Becke-Johnson damping, D3(BJ), and (ii) the aug-cc-pVTZ basis sets were used.

Method	Δ*E*_el_^‡^/cm^−1^	Δ*E*_0_^‡^/cm^−1^	$\tilde{v}\tilde{v}$_imag_/i cm^−1^
B3LYP	118	117	32.0
PBE0	112	112	31.3
CAM-B3LYP	112	112	31.7
B2PLYP	127	126	33.0
DSD-PBEP86-D3	139	138	34.2

**Table 5:** Key structural parameters for hexafluoroacetone imine determined from the zero-point corrected PBE0-D3(BJ)/aug-cc-pVTZ structure. See Figure 3 for the atom labeling scheme.

Parameter	Value
*r* (C_1_-C_3_)/Å	1.534
*r* (C_2_-C_3_)/Å	1.536
*r* (C_3_-N)/Å	1.984
∠ (C_1_-C_3_-C_2_)/degrees	116.4
*D*(F_1_-C_1_-C_3_-N)/degrees	−20.5

**Table 6:** The PBE0-D3(BJ)/aug-cc-pVTZ equilibrium X_e_, and zero-point corrected X_0_ rotational constants for hexafluoroacetone imine. All values are in MHz.

	*A*_e_	*A*_0_	*B*_e_	*B*_0_	*C*_e_	*C*_0_
Parent	2180.96	2168.545	1045.109	1038.330	938.792	933.093
^13^C=N	2175.995	2163.619	1045.109	1038.444	937.871	932.272
^15^N	2145.054	2132.83	1045.102	1038.319	932.072	926.398
^13^C, N lone pair side	2180.916	2168.619	1041.447	1034.739	935.829	930.183
^13^C, NH side	2180.895	2168.594	1041.510	1034.811	935.875	930.243

**Table 7:** Kraitchman substitution coordinates, in Angstroms, for the heavy atoms in hexafluoroacetone imine. The *c*-coordinates are either imaginary * or are zero to two significant figures.

	State I		State II		PBE0-D3(BJ)/aug-cc-pVTZ	
	*a*	*b*	*a*	*b*	*a*	*b*
C_1_	−1.2867(12)	0 *	−1.2876(12)	0 *	−1.29	−0.08
C_2_	1.2968(11)	0 *	1.2980(12)	0 *	1.30	0.07
C_3_	0 *	0.7244(21)	0 *	0.7229(21)	0	0.73
N	0.060(25)	1.9910(7)	0.073(20)	1.9904(7)	0.06	1.98

* These coordinates are imaginary consistent with the atom being close, or on, a principal axis. We have taken them to indicate a coordinate of zero.

**Table 8:** The values of the ^14^N-nuclear quadrupole coupling tensor components, χ, for hexafluoroacetone imine, methanimine (CH_2_NH) [24] and difluomethanimine (CF_2_NH) [25,26].

	CH_2_NH	CF_2_NH	(CF_3_)_2_CNH
χ_aa/_MHz	−0.9131(16)	1.029(20)	−5.0732(33)
χ_bb_/MHz	−2.6688(14)	−2.56(17)	2.9924(40)
χ_cc_/MHz	3.5819(21)	1.531(22)	2.0809(40)

## Data Availability

All data presented in this study are available on request from the corresponding authors.

## Supplementary Materials

The following supporting information can be downloaded at https://www.mdpi.com/article/10.3390/molecules30092051/s1, Table S1: Transition frequencies and quantum number assignments for hexafluoroacetone imine. Table S2: Overview of the electronic ($∆E_{el}^{‡}$) and zero-point corrected ($∆E_{0}^{‡}$) barrier for the inversion. The singular imaginary mode ($\tilde{v}$_imag_) is also given associated with the transition state. Figure S1: 2-D potential energy scan for the six molecules of interest, acetone (top left), acetone imine (middle left), isobutene (bottom left), and, in each case, the hexafluorinated versions are shown to their right. Stationary points for the hexafluorinated species are shown with a white cross. See text for methods used. Figure S2: NEB scans for the fluorinated variants using ORCA 6.0.1 and 60 images and B3LYP-D3(BJ)/aVTZ (using Gaussian’s definition of B3LYP). The angle here is the average of the two dihedral angles defining the rotation of the CF3 groups.
