# Supplementary material for: A Pure Rotational Spectroscopic Study of Two Nearly-Equivalent Structures of Hexafluoroacetone Imine, (CF3)C=NH
Source: Molecules. 2025 May 5;30(9):2051. doi: 10.3390/molecules30092051 (PMC12073689; doi:10.3390/molecules30092051)
Supplement: Supplementary file 1 [file molecules-30-02051-s001.zip › molecules-3590405-supplementary.pdf]

Supplementary Information:

A Pure Rotational Spectroscopic Study of Two Nearly-Equivalent Structures of Hexafluoroacetone Imine, $(CF_3)C{=}NH$

Daniel A. Obenchain [1,*], Beppo Hartwig [1], Daniel J. Frohman [2], G. S. Grubbs II [3], B. E. Long [4], Wallace C. Pringle [2], Stewart E. Novick [2,†], and S. A. Cooke [5,*]

[1] Institute of Physical Chemistry, University of Göttingen, Tammannstrasse 6, 37077 Göttingen, Germany
[2] Department of Chemistry, Wesleyan University, Hall-Atwater Laboratories, 52 Lawn Avenue, Middletown, CT 06459-0180, USA
[3] Missouri University of Science and Technology, Department of Chemistry, 104 Schrenk Hall, 400 W. 11th St, Rolla, MO, 65409, U.S.A.; grubbsg@mst.edu
[4] Department of Chemistry and Biochemistry, James Madison University, Harrisonburg, VA 22807, U.S.A.; long2be@jmu.edu
[5] Purchase College SUNY, School of Natural and Social Sciences, 735 Anderson Hill Rd, Purchase, NY, 10577, U.S.A.
* Correspondence: daniel.obenchain@uni-goettingen.de (D.A.O), Stephen.cooke@purchase.edu (S.A.C)
† Deceased, 10/2023

Supp Data Table S1: Transition frequencies and quantum number assignments for hexafluoroacetone imine.

Spectroscopic transitions and quantum number assignments for hexafluoroacetone imine parent.

/ instead of : below denotes (o-c)>3*err

			obs	o-c	error o-c	blends wt	Notes
1:	3 1 3 1 3	2 1 2 1 2	5773.9338	0.0005	0.003		
2:	3 1 3 0 3	2 1 2 0 2	5773.9447	0.0014	0.003		
3:	3 1 3 1 2	2 1 2 1 1	5774.1053	0.0002	0.003		
4:	3 1 3 0 2	2 1 2 0 1	5774.1169	0.0018	0.003		
5:	3 1 3 1 4	2 1 2 1 3	5774.2596	-0.0004	0.003		
6:	3 1 3 0 4	2 1 2 0 3	5774.2703	0.0003	0.003		
7:	3 0 3 1 2	2 0 2 1 1	5909.9738	-0.0023	0.003		
8:	3 0 3 0 3	2 0 2 0 2	5910.0160	0.0026	0.003		
9:	3 0 3 1 4	2 0 2 1 3	5910.1398	-0.0027	0.003		
10:	3 0 3 0 4	2 0 2 0 3	5910.1630	0.0027	0.003		
11:	3 2 2 1 4	2 2 1 1 3	5939.0618	0.0006	0.003		
12:	3 2 2 0 4	2 2 1 0 3	5939.0828	-0.0001	0.003		
13:	3 2 2 1 2	2 2 1 1 1	5939.6076	-0.0032	0.003		
14:	3 2 2 0 2	2 2 1 0 1	5939.6312	-0.0015	0.003		
15:	3 2 1 1 2	2 2 0 1 2	5966.6787	-0.0002	0.003		
16:	3 2 1 0 2	2 2 0 0 2	5966.7017	-0.0028	0.003		
17:	3 2 1 1 3	2 2 0 1 2	5966.9257	0.0029	0.003		
18:	3 2 1 0 3	2 2 0 0 2	5966.9453	-0.0031	0.003		
19:	3 2 1 1 4	2 2 0 1 3	5967.8038	0.0020	0.003		
20:	3 2 1 0 4	2 2 0 0 3	5967.8263	-0.0011	0.003		
21:	3 2 1 1 3	2 2 0 1 3	5967.9812	-0.0015	0.003		
22:	3 2 1 0 3	2 2 0 0 3	5968.0052	-0.0032	0.003		
23:	3 2 1 1 2	2 2 0 1 1	5968.3227	-0.0048	0.003		
24:	3 2 1 0 2	2 2 0 0 1	5968.3495	-0.0036	0.003		
25:	3 1 2 1 3	2 1 1 1 2	6094.2590	0.0013	0.003		
26:	3 1 2 0 3	2 1 1 0 2	6094.2902	0.0004	0.003		
27:	3 1 2 1 4	2 1 1 1 3	6094.5273	0.0007	0.003		
28:	3 1 2 0 4	2 1 1 0 3	6094.5588	0.0001	0.003		
29:	3 1 2 1 2	2 1 1 1 1	6094.6868	0.0007	0.003		
30:	3 1 2 0 2	2 1 1 0 1	6094.7184	0.0001	0.003		
31:	4 0 4 1 3	3 1 3 1 2	6956.2901	-0.0011	0.003		
32:	4 0 4 0 3	3 1 3 0 2	6956.3280	-0.0006	0.003		
33:	4 0 4 1 5	3 1 3 1 4	6956.5106	-0.0004	0.003		
34:	4 0 4 0 5	3 1 3 0 4	6956.5479	-0.0004	0.003		
35:	4 0 4 1 4	3 1 3 1 3	6956.7750	0.0006	0.003		
36:	4 0 4 0 4	3 1 3 0 3	6956.8118	0.0000	0.003		
37:	2 2 1 1 2	1 1 0 1 1	7446.6195	-0.0030	0.003		

38:	2	2	1	0	2	1	1	0	0	1	7446.6268	-0.0046	0.003
39:	2	2	1	1	3	1	1	0	1	2	7446.8524	-0.0036	0.003
40:	2	2	1	0	3	1	1	0	0	2	7446.8641	-0.0008	0.003
41:	2	2	1	1	1	1	1	0	1	0	7448.1385	-0.0021	0.003
42:	2	2	1	0	1	1	1	0	0	0	7448.1456	-0.0039	0.003
43:	4	1	4	1	4	3	1	3	1	3	7690.9821	0.0004	0.003
44:	4	1	4	0	4	3	1	3	0	3	7690.9952	0.0011	0.003
45:	4	1	4	1	3	3	1	3	1	2	7691.0491	0.0005	0.003
46:	4	1	4	0	3	3	1	3	0	2	7691.0612	0.0003	0.003
47:	4	1	4	1	5	3	1	3	1	4	7691.1559	0.0000	0.003
48:	4	1	4	0	5	3	1	3	0	4	7691.1682	0.0001	0.003
49:	4	0	4	1	4	3	0	3	1	3	7847.8056	0.0004	0.003
50:	4	0	4	0	4	3	0	3	0	3	7847.8233	-0.0009	0.003
51:	4	0	4	1	3	3	0	3	1	2	7847.9035	0.0010	0.003
52:	4	0	4	0	3	3	0	3	0	2	7847.9214	0.0000	0.003
53:	4	0	4	1	5	3	0	3	1	4	7847.9718	0.0000	0.003
54:	4	0	4	0	5	3	0	3	0	4	7847.9899	-0.0009	0.003
55:	4	1	3	1	4	3	1	2	1	3	8116.6449	0.0000	0.003
56:	4	1	3	0	4	3	1	2	0	3	8116.6871	0.0012	0.003
57:	4	1	3	1	5	3	1	2	1	4	8116.7884	0.0001	0.003
58:	4	1	3	0	5	3	1	2	0	4	8116.8287	-0.0004	0.003
59:	4	1	3	1	3	3	1	2	1	2	8116.8320	0.0022	0.003
60:	4	1	3	0	3	3	1	2	0	2	8116.8773	0.0066	0.003
61:	4	1	4	0	4	3	0	3	0	3	8582.0048	-0.0016	0.003
62:	4	1	4	1	4	3	0	3	1	3	8582.0143	0.0018	0.003
63:	5	0	5	1	4	4	1	4	1	3	9027.4715	-0.0009	0.003
64:	5	0	5	0	4	4	1	4	0	3	9027.5152	-0.0002	0.003
65:	5	0	5	1	6	4	1	4	1	5	9027.6213	-0.0004	0.003
66:	5	0	5	0	6	4	1	4	0	5	9027.6647	0.0000	0.003
67:	5	0	5	1	5	4	1	4	1	4	9027.8790	-0.0003	0.003
68:	5	0	5	0	5	4	1	4	0	4	9027.9222	0.0000	0.003
69:	5	1	5	1	5	4	1	4	1	4	9602.1537	-0.0001	0.003
70:	5	1	5	0	5	4	1	4	0	4	9602.1673	-0.0004	0.003
71:	5	1	5	1	4	4	1	4	1	3	9602.2024	0.0009	0.003
72:	5	1	5	0	4	4	1	4	0	3	9602.2149	-0.0002	0.003
73:	5	1	5	1	6	4	1	4	1	5	9602.2741	0.0002	0.003
74:	5	1	5	0	6	4	1	4	0	5	9602.2874	-0.0002	0.003
75:	5	0	5	1	5	4	0	4	1	4	9762.0865	0.0000	0.003
76:	5	0	5	0	5	4	0	4	0	4	9762.1039	-0.0006	0.003
77:	5	0	5	1	4	4	0	4	1	3	9762.2298	0.0000	0.003
78:	5	0	5	0	4	4	0	4	0	3	9762.2478	0.0000	0.003
79:	5	0	5	1	6	4	0	4	1	5	9762.2670	0.0003	0.003
80:	5	0	5	0	6	4	0	4	0	5	9762.2849	0.0003	0.003
81:	5	2	4	1	5	4	2	3	1	5	9881.3314	-0.0052	0.003
82:	5	2	4	0	5	4	2	3	0	5	9881.3739	0.0036	0.003
83:	5	2	4	1	5	4	2	3	1	4	9881.7703	-0.0035	0.003

84:	5 2 4 0 5	4 2 3 0 4	9881.8119	0.0045	0.003
85:	5 2 4 1 6	4 2 3 1 5	9882.0176	-0.0038	0.003
86:	5 2 4 1 4	4 2 3 1 3	9882.0520	0.0034	0.003
87:	5 2 4 0 6	4 2 3 0 5	9882.0597	0.0045	0.003
88:	5 2 4 0 4	4 2 3 0 3	9882.0867	0.0045	0.003
89:	5 2 4 1 4	4 2 3 1 4	9882.5921	-0.0063	0.003
90:	5 2 4 0 4	4 2 3 0 4	9882.6352	0.0031	0.003
91:	5 4 2 1 5	4 4 1 1 4	9913.5361	0.0023	0.003
92:	5 4 2 0 5	4 4 1 0 4	9913.5735	0.0016	0.003
93:	5 4 1 1 5	4 4 0 1 4	9913.6197	-0.0009	0.003
94:	5 4 1 0 5	4 4 0 0 4	9913.6564	-0.0024	0.003
95:	5 4 2 1 6	4 4 1 1 5	9914.3372	0.0017	0.003
96:	5 4 1 1 6	4 4 0 1 5	9914.4188	-0.0033	0.003
97:	5 4 1 0 6	4 4 0 0 5	9914.4564	-0.0038	0.003
98:	5 4 2 1 4	4 4 1 1 3	9914.5774	-0.0009	0.003
99:	5 4 2 0 4	4 4 1 0 3	9914.6209	0.0044	0.003
100:	5 4 1 0 4	4 4 0 0 3	9914.7012	-0.0018	0.003
101/	5 3 3 1 5	4 3 2 1 4	9919.5556	0.0116	0.003
102:	5 3 3 0 5	4 3 2 0 4	9919.5859	0.0035	0.003
103/	5 3 3 1 6	4 3 2 1 5	9920.0043	0.0118	0.003
104:	5 3 3 0 6	4 3 2 0 5	9920.0341	0.0032	0.003
105/	5 3 3 1 4	4 3 2 1 3	9920.1182	0.0118	0.003
106:	5 3 3 0 4	4 3 2 0 3	9920.1478	0.0030	0.003
107:	5 3 2 1 5	4 3 1 1 4	9925.9614	-0.0025	0.003
108/	5 3 2 0 5	4 3 1 0 4	9925.9932	-0.0103	0.003
109:	5 3 2 1 6	4 3 1 1 5	9926.3927	-0.0032	0.003
110/	5 3 2 0 6	4 3 1 0 5	9926.4241	-0.0114	0.003
111:	5 3 2 1 4	4 3 1 1 3	9926.5048	-0.0022	0.003
112/	5 3 2 0 4	4 3 1 0 3	9926.5367	-0.0099	0.003
113:	5 2 3 1 5	4 2 2 1 4	10017.6615	0.0005	0.003
114:	5 2 3 0 5	4 2 2 0 4	10017.7089	-0.0019	0.003
115:	5 2 3 1 6	4 2 2 1 5	10017.7474	0.0022	0.003
116:	5 2 3 0 6	4 2 2 0 5	10017.7956	0.0006	0.003
117:	5 1 4 1 5	4 1 3 1 4	10130.1222	0.0011	0.010
118:	5 1 4 0 5	4 1 3 0 4	10130.1710	0.0020	0.010
119:	5 1 4 1 6	4 1 3 1 5	10130.2320	0.0010	0.010
120:	5 1 4 1 4	4 1 3 1 3	10130.2486	0.0001	0.010
121:	5 1 4 0 6	4 1 3 0 5	10130.2798	0.0009	0.010
122:	5 1 4 0 4	4 1 3 0 3	10130.2995	0.0031	0.010
123:	5 1 5 0 5	4 0 4 0 5	10334.9772	0.0008	0.003
124:	5 1 5 1 5	4 0 4 1 5	10334.9851	-0.0025	0.003
125:	5 1 5 0 5	4 0 4 0 4	10336.3523	0.0023	0.003
126:	5 1 5 1 5	4 0 4 1 4	10336.3617	0.0004	0.003
127:	5 1 5 0 6	4 0 4 0 5	10336.9135	0.0060	0.003
128:	5 1 5 1 6	4 0 4 1 5	10336.9196	0.0008	0.003
129:	5 1 5 0 4	4 0 4 0 3	10336.9497	0.0022	0.003

130:	5	1	5	1	4	4	0	4	1	3	10336.9592	0.0004	0.003
131:	5	1	5	0	4	4	0	4	0	4	10338.6810	0.0066	0.003
132:	5	1	5	1	4	4	0	4	1	4	10338.6888	0.0031	0.003
133:	6	0	6	1	6	5	1	5	1	6	11077.7340	-0.0037	0.003
134:	6	0	6	0	6	5	1	5	0	6	11077.7746	-0.0074	0.003
135:	6	0	6	1	5	5	1	5	1	4	11079.3685	-0.0014	0.003
136:	6	0	6	0	5	5	1	5	0	4	11079.4145	0.0002	0.003
137:	6	0	6	1	7	5	1	5	1	6	11079.4699	-0.0007	0.003
138:	6	0	6	0	7	5	1	5	0	6	11079.5156	0.0005	0.003
139:	6	0	6	1	6	5	1	5	1	5	11079.6678	-0.0009	0.003
140:	6	0	6	0	6	5	1	5	0	5	11079.7137	0.0005	0.003
141:	4	2	3	0	4	3	1	2	0	3	11137.5759	-0.0077	0.003
142:	4	2	3	1	4	3	1	2	1	3	11137.5842	0.0053	0.003
143:	4	2	3	0	5	3	1	2	0	4	11138.0660	-0.0068	0.003
144:	4	2	3	1	5	3	1	2	1	4	11138.0744	0.0062	0.003
145:	4	2	3	0	3	3	1	2	0	2	11138.1962	-0.0070	0.003
146:	4	2	3	1	3	3	1	2	1	2	11138.2044	0.0059	0.003
147:	6	1	6	1	6	5	1	5	1	5	11507.0323	-0.0004	0.003
148:	6	1	6	0	6	5	1	5	0	5	11507.0470	-0.0003	0.003
149:	6	1	6	1	7	5	1	5	1	6	11507.1279	-0.0001	0.003
150:	6	1	6	0	7	5	1	5	0	6	11507.1425	-0.0002	0.003
151:	6	1	6	1	5	5	1	5	1	5	11509.3969	-0.0044	0.003
152:	6	1	6	0	5	5	1	5	0	5	11509.4191	0.0030	0.003
153:	6	0	6	1	6	5	0	5	1	5	11653.9436	0.0001	0.003
154:	6	0	6	0	6	5	0	5	0	5	11653.9583	-0.0002	0.003
155:	6	0	6	1	5	5	0	5	1	4	11654.0992	0.0003	0.003
156:	6	0	6	0	5	5	0	5	0	4	11654.1130	-0.0010	0.003
157:	6	0	6	1	7	5	0	5	1	6	11654.1236	0.0008	0.003
158:	6	0	6	0	7	5	0	5	0	6	11654.1378	-0.0001	0.003
159:	3	3	1	0	2	2	2	0	0	1	11837.8472	0.0003	0.010
160:	3	3	1	1	3	2	2	0	1	2	11837.9377	-0.0062	0.010
161:	3	3	1	0	3	2	2	0	0	2	11837.9595	0.0005	0.010
162:	6	2	5	1	6	5	2	4	1	5	11845.0129	-0.0035	0.003
163:	6	2	5	0	6	5	2	4	0	5	11845.0590	0.0043	0.003
164:	6	2	5	1	7	5	2	4	1	6	11845.1786	-0.0045	0.003
165:	6	2	5	1	5	5	2	4	1	4	11845.1843	-0.0031	0.003
166:	6	2	5	0	7	5	2	4	0	6	11845.2247	0.0034	0.003
167:	6	2	5	0	5	5	2	4	0	4	11845.2304	0.0049	0.003
168:	6	3	4	1	6	5	3	3	1	5	11909.2258	0.0019	0.003
169:	6	3	4	0	6	5	3	3	0	5	11909.2711	0.0015	0.003
170:	6	3	4	1	7	5	3	3	1	6	11909.4829	0.0013	0.003
171:	6	3	4	0	7	5	3	3	0	6	11909.5278	0.0005	0.003
172:	6	3	4	0	5	5	3	3	0	4	11909.5718	0.0020	0.003
173:	6	1	5	1	6	5	1	4	1	5	12131.3809	0.0001	0.003
174:	6	1	5	0	6	5	1	4	0	5	12131.4346	0.0012	0.003
175:	6	1	5	1	7	5	1	4	1	6	12131.4857	-0.0009	0.003

176:	6	1	5	1	5	5	1	4	1	4	12131.4973	0.0000	0.003
177:	6	1	5	0	7	5	1	4	0	6	12131.5395	0.0002	0.003
178:	6	1	5	0	5	5	1	4	0	4	12131.5516	0.0015	0.003
179:	7	0	7	1	7	6	1	6	1	7	13099.4247	0.0009	0.003
180:	7	0	7	0	7	6	1	6	0	7	13099.4616	-0.0040	0.003
181:	7	0	7	1	6	6	1	6	1	5	13101.2525	-0.0001	0.003
182:	7	0	7	0	6	6	1	6	0	5	13101.2944	-0.0002	0.003
183:	7	0	7	1	8	6	1	6	1	7	13101.3209	-0.0002	0.003
184:	7	0	7	0	8	6	1	6	0	7	13101.3630	0.0000	0.003
185:	7	0	7	1	7	6	1	6	1	6	13101.4499	-0.0001	0.003
186:	7	0	7	0	7	6	1	6	0	6	13101.4915	-0.0005	0.003
187:	7	1	7	1	7	6	1	6	1	6	13405.6477	0.0002	0.003
188:	7	1	7	0	7	6	1	6	0	6	13405.6627	0.0003	0.003
189:	7	1	7	1	6	6	1	6	1	5	13405.6894	-0.0017	0.003
190:	7	1	7	0	6	6	1	6	0	5	13405.7054	-0.0006	0.003
191:	7	1	7	1	8	6	1	6	1	7	13405.7295	0.0002	0.003
192:	7	1	7	0	8	6	1	6	0	7	13405.7449	0.0007	0.003
193:	7	0	7	1	7	6	0	6	1	6	13528.8144	0.0003	0.003
194:	7	0	7	0	7	6	0	6	0	6	13528.8258	-0.0005	0.003
195:	7	0	7	1	6	6	0	6	1	5	13528.9601	0.0005	0.003
196:	7	0	7	0	6	6	0	6	0	5	13528.9700	-0.0018	0.003
197:	7	0	7	1	8	6	0	6	1	7	13528.9799	0.0013	0.003
198:	7	0	7	0	8	6	0	6	0	7	13528.9902	-0.0005	0.003
199:	7	2	6	1	7	6	2	5	1	7	13800.3587	-0.0001	0.003
200:	7	2	6	0	7	6	2	5	0	7	13800.3980	-0.0023	0.003
201:	7	2	6	1	7	6	2	5	1	6	13801.2110	0.0006	0.003
202:	7	2	6	0	7	6	2	5	0	6	13801.2513	-0.0005	0.003
203:	4	3	1	1	3	3	2	2	1	2	13840.1711	-0.0063	0.003
204:	4	3	1	0	3	3	2	2	0	2	13840.2088	0.0060	0.003
205:	4	3	1	1	5	3	2	2	1	4	13840.2836	-0.0050	0.003
206:	4	3	1	0	5	3	2	2	0	4	13840.3209	0.0070	0.003
207:	4	3	1	1	4	3	2	2	1	3	13840.7130	-0.0072	0.003
208:	4	3	1	0	4	3	2	2	0	3	13840.7510	0.0055	0.003
209:	7	3	5	1	7	6	3	4	1	7	13899.3371	-0.0005	0.003
210:	7	3	5	0	7	6	3	4	0	7	13899.3886	-0.0014	0.003
211:	7	3	5	1	7	6	3	4	1	6	13899.6231	0.0009	0.003
212:	7	3	5	0	7	6	3	4	0	6	13899.6746	0.0000	0.003
213:	7	3	5	1	8	6	3	4	1	7	13899.7849	0.0005	0.003
214:	7	3	5	1	6	6	3	4	1	5	13899.8026	0.0019	0.003
215:	7	3	5	0	8	6	3	4	0	7	13899.8363	-0.0004	0.003
216:	7	3	5	0	6	6	3	4	0	5	13899.8537	0.0005	0.003
217:	7	3	5	1	6	6	3	4	1	6	13900.1319	-0.0015	0.003
218:	7	3	5	0	6	6	3	4	0	6	13900.1835	-0.0023	0.003
219:	7	3	4	1	7	6	3	3	1	6	13936.9136	0.0001	0.003
220:	7	3	4	0	7	6	3	3	0	6	13936.9714	-0.0005	0.003
221:	7	3	4	1	8	6	3	3	1	7	13937.0279	0.0000	0.003

222:	7	3	4	1	6	6	3	3	1	5	13937.0397	0.0008	0.003
223:	7	3	4	0	8	6	3	3	0	7	13937.0859	-0.0005	0.003
224:	7	3	4	0	6	6	3	3	0	5	13937.0983	0.0009	0.003
225:	7	1	6	1	7	6	1	5	1	6	14116.4737	0.0002	0.003
226:	7	1	6	0	7	6	1	5	0	6	14116.5271	-0.0010	0.003
227:	7	1	6	1	8	6	1	5	1	7	14116.5883	0.0002	0.003
228:	7	1	6	1	6	6	1	5	1	5	14116.5985	0.0015	0.003
229:	7	1	6	0	8	6	1	5	0	7	14116.6421	-0.0007	0.003
230:	7	1	6	0	6	6	1	5	0	5	14116.6525	0.0007	0.003
231:	7	2	5	1	6	6	2	4	1	5	14128.3219	-0.0003	0.003
232:	7	2	5	1	8	6	2	4	1	7	14128.3326	0.0019	0.003
233:	7	2	5	1	7	6	2	4	1	6	14128.3560	0.0000	0.003
234:	7	2	5	0	6	6	2	4	0	5	14128.3946	-0.0011	0.003
235:	7	2	5	0	8	6	2	4	0	7	14128.4052	0.0011	0.003
236:	7	2	5	0	7	6	2	4	0	6	14128.4291	-0.0003	0.003
237:	5	2	3	1	4	4	1	4	1	3	14211.2420	0.0014	0.003
238:	5	2	3	0	4	4	1	4	0	3	14211.3424	0.0011	0.003
239:	5	2	3	1	6	4	1	4	1	5	14211.6674	0.0015	0.003
240:	5	2	3	0	6	4	1	4	0	5	14211.7673	0.0007	0.003
241:	8	0	8	1	7	7	1	7	1	6	15089.7436	-0.0004	0.003
242:	8	0	8	0	7	7	1	7	0	6	15089.7812	-0.0003	0.003
243:	8	0	8	1	9	7	1	7	1	8	15089.7920	0.0005	0.003
244:	8	0	8	0	9	7	1	7	0	8	15089.8291	0.0002	0.003
245:	8	0	8	1	8	7	1	7	1	7	15089.8604	-0.0007	0.003
246:	8	0	8	0	8	7	1	7	0	7	15089.8975	-0.0010	0.003
247:	8	1	8	1	8	7	1	7	1	7	15298.4400	0.0000	0.003
248:	8	1	8	0	8	7	1	7	0	7	15298.4538	-0.0011	0.003
249:	8	1	8	1	7	7	1	7	1	6	15298.4835	0.0003	0.003
250:	8	1	8	0	7	7	1	7	0	6	15298.4990	0.0009	0.003
251:	8	1	8	1	9	7	1	7	1	8	15298.5132	0.0006	0.003
252:	8	1	8	0	9	7	1	7	0	8	15298.5271	-0.0003	0.003
253:	8	0	8	1	8	7	0	7	1	8	15392.1568	-0.0043	0.003
254:	8	0	8	0	8	7	0	7	0	8	15392.1731	0.0016	0.003
255:	8	0	8	1	8	7	0	7	1	7	15394.0576	-0.0009	0.003
256:	8	0	8	0	8	7	0	7	0	7	15394.0697	0.0008	0.003
257:	8	0	8	1	7	7	0	7	1	6	15394.1813	-0.0012	0.003
258:	8	0	8	0	7	7	0	7	0	6	15394.1923	-0.0005	0.003
259:	8	0	8	1	9	7	0	7	1	8	15394.2002	0.0005	0.003
260:	8	0	8	0	9	7	0	7	0	8	15394.2116	0.0017	0.003
261:	8	0	8	1	7	7	0	7	1	7	15396.3488	-0.0049	0.003
262:	8	1	8	0	8	7	0	7	0	7	15602.6249	-0.0003	0.003
263:	8	1	8	1	8	7	0	7	1	7	15602.6383	0.0008	0.003
264:	8	1	8	0	9	7	0	7	0	8	15602.9088	0.0002	0.003
265:	8	1	8	0	7	7	0	7	0	6	15602.9094	-0.0001	0.003
266:	8	1	8	1	9	7	0	7	1	8	15602.9219	0.0011	0.003
267:	8	2	7	1	8	7	2	6	1	7	15749.4639	0.0000	0.003

268:	8	2	7	0	8	7	2	6	0	7	15749.5059	-0.0019	0.003
269:	8	2	7	1	7	7	2	6	1	6	15749.5673	0.0002	0.003
270:	8	2	7	1	9	7	2	6	1	8	15749.5732	0.0018	0.003
271:	8	2	7	0	7	7	2	6	0	6	15749.6100	-0.0010	0.003
272:	8	2	7	0	9	7	2	6	0	8	15749.6152	-0.0001	0.003
273:	8	3	6	1	8	7	3	5	1	7	15889.4350	0.0006	0.003
274:	8	3	6	0	8	7	3	5	0	7	15889.4916	-0.0011	0.003
275:	8	3	6	1	9	7	3	5	1	8	15889.5462	0.0006	0.003
276:	8	3	6	1	7	7	3	5	1	6	15889.5535	0.0022	0.003
277:	8	3	6	0	9	7	3	5	0	8	15889.6029	-0.0009	0.003
278:	8	3	6	0	7	7	3	5	0	6	15889.6100	0.0002	0.003
279:	8	3	6	1	7	7	3	5	1	8	15889.6167	0.0007	0.003
280:	8	3	6	0	7	7	3	5	0	8	15889.6747	0.0004	0.003
281:	8	4	4	1	9	7	4	3	1	8	15889.8120	-0.0042	0.003
282:	8	4	4	0	9	7	4	3	0	8	15889.8789	0.0024	0.003
283:	8	3	5	1	8	7	3	4	1	7	15961.6906	-0.0009	0.003
284:	8	3	5	0	8	7	3	4	0	7	15961.7602	-0.0006	0.003
285:	8	1	7	1	8	7	1	6	1	7	16081.1154	-0.0002	0.003
286:	8	1	7	0	8	7	1	6	0	7	16081.1692	0.0000	0.003
287:	8	1	7	1	9	7	1	6	1	8	16081.2447	-0.0004	0.003
288:	8	1	7	1	7	7	1	6	1	6	16081.2544	0.0007	0.003
289:	8	1	7	0	9	7	1	6	0	8	16081.2983	-0.0005	0.003
290:	8	1	7	0	7	7	1	6	0	6	16081.3077	0.0003	0.003
291:	8	2	6	1	7	7	2	5	1	6	16188.6932	-0.0009	0.003
292:	8	2	6	1	9	7	2	5	1	8	16188.7015	0.0014	0.003
293:	8	2	6	1	8	7	2	5	1	7	16188.7198	-0.0012	0.003
294:	8	2	6	0	7	7	2	5	0	6	16188.7744	-0.0009	0.003
295:	8	2	6	0	9	7	2	5	0	8	16188.7829	0.0015	0.003
296:	8	2	6	0	8	7	2	5	0	7	16188.8021	-0.0001	0.003
297:	9	1	9	1	9	8	1	8	1	9	17183.9651	-0.0026	0.003
298:	9	1	9	0	9	8	1	8	0	9	17183.9841	0.0017	0.003
299:	9	1	9	1	9	8	1	8	1	8	17186.1488	0.0002	0.003
300:	9	1	9	1	8	8	1	8	1	7	17186.1869	-0.0034	0.003
301:	9	1	9	0	8	8	1	8	0	7	17186.2038	-0.0011	0.003
302:	9	1	9	1	10	8	1	8	1	9	17186.2142	0.0002	0.003
303:	9	1	9	1	8	8	1	8	1	8	17188.6423	-0.0035	0.003
304:	9	1	9	0	8	8	1	8	0	8	17188.6596	-0.0008	0.003
305:	9	0	9	1	9	8	0	8	1	8	17255.9610	0.0000	0.003
306:	9	0	9	0	9	8	0	8	0	8	17255.9716	0.0010	0.003
307:	9	0	9	1	8	8	0	8	1	7	17256.0592	-0.0016	0.003
308:	9	0	9	0	8	8	0	8	0	7	17256.0695	-0.0009	0.003
309:	9	0	9	1	10	8	0	8	1	9	17256.0776	0.0008	0.003
310:	9	0	9	0	10	8	0	8	0	9	17256.0878	0.0014	0.003
311:	9	1	9	0	9	8	0	8	0	8	17394.7189	-0.0006	0.003
312:	9	1	9	1	9	8	0	8	1	8	17394.7290	0.0016	0.003
313:	9	1	9	0	10	8	0	8	0	9	17394.9206	-0.0065	0.003

```
314:  9  1  9  1 10    8  0  8  1  9       17394.9372   0.0021  0.003
315: 10  1 10  1 10    9  1  9  1 10       19067.4206   0.0032  0.003
316: 10  1 10  0 10    9  1  9  0 10       19067.4347   0.0031  0.003
317: 10  1 10  1 10    9  1  9  1  9       19069.6639   0.0004  0.003
318: 10  1 10  0 10    9  1  9  0  9       19069.6785   0.0008  0.003
319: 10  1 10  1  9    9  1  9  1  8       19069.7037   0.0008  0.003
320: 10  1 10  1 11    9  1  9  1 10       19069.7251   0.0027  0.003
321: 10  1 10  0 11    9  1  9  0 10       19069.7364  -0.0001  0.003
322: 10  0 10  1 10    9  0  9  1  9       19118.2887  -0.0008  0.003
323: 10  0 10  0 10    9  0  9  0  9       19118.2994   0.0001  0.003
324: 10  0 10  1  9    9  0  9  1  8       19118.3684   0.0004  0.003
325: 10  0 10  0  9    9  0  9  0  8       19118.3770  -0.0006  0.003
326: 10  0 10  1 11    9  0  9  1 10       19118.3844   0.0014  0.003
327: 10  0 10  0 11    9  0  9  0 10       19118.3938   0.0011  0.003
328:  6  4  3  1  5    5  3  2  1  4       20137.9425   0.0058  0.003
329:  6  4  3  0  5    5  3  2  0  4       20137.9583  -0.0047  0.003
330: 10  6  5  1 10    9  6  4  1  9       19842.3788  -0.0006  0.003
331: 10  6  4  1 10    9  6  3  1  9       19842.3911  -0.0023  0.003
332: 10  6  5  0 10    9  6  4  0  9       19842.4508   0.0014  0.003
333: 10  6  4  0 10    9  6  3  0  9       19842.4628  -0.0005  0.003
334: 10  6  5  1 11    9  6  4  1 10       19842.6018  -0.0016  0.003
335: 10  6  4  1 11    9  6  3  1 10       19842.6134  -0.0040  0.003
336: 10  6  5  1  9    9  6  4  1  8       19842.6315   0.0023  0.003
337: 10  6  4  1  9    9  6  3  1  8       19842.6434   0.0003  0.003
338: 10  6  5  0 11    9  6  4  0 10       19842.6726  -0.0008  0.003
339: 10  6  4  0 11    9  6  3  0 10       19842.6853  -0.0021  0.003
340: 10  6  5  0  9    9  6  4  0  8       19842.7024   0.0033  0.003
341: 10  6  4  0  9    9  6  3  0  8       19842.7144   0.0013  0.003
342: 10  4  7  1 10    9  4  6  1  9       19881.1357   0.0018  0.003
343: 10  4  7  0 10    9  4  6  0  9       19881.2064   0.0009  0.003
344: 10  4  7  1 11    9  4  6  1 10       19881.2217   0.0004  0.003
345: 10  4  7  1  9    9  4  6  1  8       19881.2276   0.0018  0.003
346: 10  4  7  0 11    9  4  6  0 10       19881.2912  -0.0016  0.003
347: 10  4  7  0  9    9  4  6  0  8       19881.2970  -0.0003  0.003
348:  4  2  2  1  3    3  1  3  1  2       11884.5340   0.0010  0.003
349:  4  2  2  0  3    3  1  3  0  2       11884.5965   0.0004  0.003
350:  4  2  2  1  5    3  1  3  1  4       11885.0773   0.0007  0.003
351:  4  2  2  0  5    3  1  3  0  4       11885.1403   0.0006  0.003
352:  4  2  2  1  4    3  1  3  1  3       11886.6014   0.0008  0.003
353:  4  2  2  0  4    3  1  3  0  3       11886.6644   0.0007  0.003
-------------------------------------------------------------------------------
```

PARAMETERS IN FIT (values truncated and Nlines statistics):

```
    10000        A  /MHz       2170.17085(35)              1
```

10011	A /MHz	2170.16744(35)	2
20000	B /MHz	1043.25444(14)	3
20011	B /MHz	1043.24680(14)	4
30000	C /MHz	936.37029(13)	5
30011	C /MHz	936.37059(13)	6
200	Del_J /kHz	0.0576(10)	7
211	Del_J /kHz	0.0525(10)	8
1100	Del_JK /kHz	0.2047(62)	9
1111	Del_JK /kHz	0.2219(62)	10
2000	Del_K /kHz	-0.362(26)	11
2011	Del_K /kHz	-0.457(26)	12
40100	del_J /kHz	0.00734(44)	13
40111	del_J /kHz	0.00471(43)	14
41000	del_K /kHz	-0.475(41)	15
41011	del_K /kHz	-0.563(41)	16
110010000	Xaa /MHz	-5.0732(33)	17
-110010011	Xaa /MHz	-5.0732(33)	= 1.00000 * 17
-110020000	Xaa /MHz	-5.0732(33)	= 1.00000 * 17
-110020011	Xaa /MHz	-5.0732(33)	= 1.00000 * 17
110030000	Xcc /MHz	2.0809(40)	18
-110030011	Xcc /MHz	2.0809(40)	= 1.00000 * 18
-110020000	Xcc /MHz	2.0809(40)	= 1.00000 * 18
-110020011	Xcc /MHz	2.0809(40)	= 1.00000 * 18

MICROWAVE AVG = -0.000014 MHz, IR AVG = 0.00000
MICROWAVE RMS = 0.002745 MHz, IR RMS = 0.00000
END OF ITERATION 1 OLD, NEW RMS ERROR= 0.90588 0.90588

distinct frequency lines in fit: 353
distinct parameters of fit: 18

MICROWAVE	lines fitted			lines	lines	RMS	RMS ERROR	J range		Ka range		freq. range	
	total	dv=0	dv.ne.0	UNFITTD	e>900								
v"= 0	176	176	0	0	0	0.002752	0.91255	1	10	0	6	5774	20138
v"= 1	177	177	0	0	0	0.002677	0.87936	1	10	0	6	5774	20138
total:	353	353	0	0	0	0.002715	0.89606						

NOTE: the RMS values above are for Nlines statistics, but the 'total' values may differ slightly from those in the .FIT file since the o-c values for this evaluation are as rounded in the .FIT.

PARAMETERS IN FIT WITH STANDARD ERRORS ON THOSE THAT ARE FITTED:
(values rounded and degrees of freedom, Ndegf=Nlines-Nconst, statistics)

10000	A /MHz	2170.17085(33)	1
10011	A /MHz	2170.16744(33)	2
20000	B /MHz	1043.25444(13)	3
20011	B /MHz	1043.24681(13)	4
30000	C /MHz	936.37030(12)	5
30011	C /MHz	936.37059(12)	6
200	Del_J /kHz	0.05761(96)	7
211	Del_J /kHz	0.05252(96)	8
1100	Del_JK /kHz	0.2047(58)	9
1111	Del_JK /kHz	0.2219(58)	10
2000	Del_K /kHz	-0.363(24)	11
2011	Del_K /kHz	-0.457(24)	12
40100	del_J /kHz	0.00734(41)	13
40111	del_J /kHz	0.00471(40)	14
41000	del_K /kHz	-0.475(38)	15
41011	del_K /kHz	-0.563(38)	16

110010000	Xaa /MHz	-5.0732(31)	17
-110010011	Xaa /MHz	-5.0732(31)	= 1.00000 * 17
-110020000	Xaa /MHz	-5.0732(31)	= 1.00000 * 17
-110020011	Xaa /MHz	-5.0732(31)	= 1.00000 * 17
110030000	Xcc /MHz	2.0809(37)	18
-110030011	Xcc /MHz	2.0809(37)	= 1.00000 * 18
-110020000	Xcc /MHz	2.0809(37)	= 1.00000 * 18
-110020011	Xcc /MHz	2.0809(37)	= 1.00000 * 18

CORRELATION COEFFICIENTS, C.ij:

	A	A	B	B	C	C	-Del_J	-Del_J
A	1.0000							
A	0.0018	1.0000						
B	0.0118	-0.0009	1.0000					
B	-0.0012	0.0070	0.0039	1.0000				
C	-0.0805	0.0012	-0.7524	-0.0032	1.0000			
C	0.0013	-0.0763	-0.0031	-0.7525	0.0027	1.0000		
-Del_J	0.0153	0.0007	-0.8652	-0.0007	0.6419	0.0006	1.0000	
-Del_J	0.0010	0.0177	-0.0010	-0.8675	0.0010	0.6458	0.0004	1.0000
-Del_JK	-0.0047	-0.0008	0.7700	0.0003	-0.7347	-0.0004	-0.9324	-0.0004
-Del_JK	-0.0010	-0.0068	0.0006	0.7712	-0.0008	-0.7366	-0.0004	-0.9326
-Del_K	-0.7897	-0.0018	-0.2178	0.0012	0.2257	-0.0013	0.2248	-0.0011
-Del_K	-0.0017	-0.7882	0.0006	-0.2147	-0.0010	0.2225	-0.0007	0.2233
-del_J	0.0450	0.0006	-0.8447	-0.0047	0.6880	0.0037	0.7173	0.0009
-del_J	0.0008	0.0468	-0.0043	-0.8498	0.0034	0.6861	0.0005	0.7335
-del_K	-0.0576	0.0014	-0.7571	-0.0010	0.8359	0.0010	0.8668	0.0008
-del_K	0.0014	-0.0552	-0.0009	-0.7578	0.0011	0.8388	0.0006	0.8666
Xaa	-0.0249	-0.0269	-0.0316	-0.0283	0.0135	0.0164	-0.0081	-0.0092
Xcc	-0.0387	-0.0420	0.0028	0.0122	-0.0157	-0.0172	-0.0151	-0.0221

	-Del_JK	-Del_JK	-Del_K	-Del_K	-del_J	-del_J	-del_K	-del_K
-Del_JK	1.0000							
-Del_JK	0.0004	1.0000						
-Del_K	-0.2564	0.0010	1.0000					
-Del_K	0.0007	-0.2551	0.0017	1.0000				
-del_J	-0.6811	-0.0005	0.1658	-0.0003	1.0000			
-del_J	-0.0001	-0.6937	-0.0008	0.1681	0.0054	1.0000		
-del_K	-0.8962	-0.0008	0.2397	-0.0013	0.5416	0.0006	1.0000	
-del_K	-0.0006	-0.8960	-0.0014	0.2377	0.0007	0.5507	0.0011	1.0000
Xaa	0.0140	0.0135	0.0264	0.0274	0.0488	0.0441	-0.0193	-0.0176
Xcc	0.0181	0.0233	0.0401	0.0401	0.0085	0.0014	-0.0300	-0.0312

	Xaa	Xcc

Xaa	1.0000	
Xcc	0.8154	1.0000

Mean value of |C.ij|, i.ne.j = 0.1921
Mean value of C.ij, i.ne.j = -0.0193

No correlations with absolute value greater than 0.9950

Worst fitted lines (obs-calc/error):

105: 3.9	103: 3.9	101: 3.9	110: -3.8
108: -3.4	112: -3.3	141: -2.6	134: -2.5
207: -2.4	206: 2.3	145: -2.3	143: -2.3
131: 2.2	60: 2.2	313: -2.2	89: -2.1
203: -2.1	144: 2.1	204: 2.0	127: 2.0
146: 2.0	328: 1.9	208: 1.8	142: 1.8
81: -1.7	205: -1.7	261: -1.6	167: 1.6
23: -1.6	329: -1.6	38: -1.5	84: 1.5
88: 1.5	164: -1.5	87: 1.5	151: -1.5
99: 1.5	253: -1.4	163: 1.4	281: -1.4
335: -1.3	180: -1.3	42: -1.3	97: -1.3
85: -1.3	133: -1.2	24: -1.2	39: -1.2
82: 1.2	162: -1.2		

```
105/ 5 3 3 1 4   4 3 2 1 3     9920.1182   0.0118  0.003
103/ 5 3 3 1 6   4 3 2 1 5     9920.0043   0.0118  0.003
101/ 5 3 3 1 5   4 3 2 1 4     9919.5556   0.0116  0.003
110/ 5 3 2 0 6   4 3 1 0 5     9926.4241  -0.0114  0.003
108/ 5 3 2 0 5   4 3 1 0 4     9925.9932  -0.0103  0.003
112/ 5 3 2 0 4   4 3 1 0 3     9926.5367  -0.0099  0.003
141: 4 2 3 0 4   3 1 2 0 3    11137.5759  -0.0077  0.003
134: 6 0 6 0 6   5 1 5 0 6    11077.7746  -0.0074  0.003
207: 4 3 1 1 4   3 2 2 1 3    13840.7130  -0.0072  0.003
206: 4 3 1 0 5   3 2 2 0 4    13840.3209   0.0070  0.003
```

/ SPFIT output reformatted with PIFORM

Spectroscopic transitions and quantum number assignments for hexafluoroacetone imine C(1)-13.

```
------------------------------------------------------------------------------=========
                                 obs        o-c    error    blends    Notes
                                                    o-c     wt
  / instead of : below denotes (o-c)>3*err
------------------------------------------------------------------------------=========
  1:  7 1 7 1 7   6 1 6 1 6     13364.3276  -0.0001  0.003
  2:  7 1 7 0 7   6 1 6 0 6     13364.3450   0.0007  0.003
  3:  7 1 7 1 6   6 1 6 1 5     13364.3705  -0.0004  0.003
  4:  7 1 7 0 6   6 1 6 0 5     13364.3886   0.0010  0.003
  5:  7 1 7 1 8   6 1 6 1 7     13364.4087  -0.0002  0.003
  6:  7 1 7 0 8   6 1 6 0 7     13364.4253  -0.0001  0.003
  7:  8 1 8 1 8   7 1 7 1 7     15251.4615   0.0006  0.003
  8:  8 1 8 0 8   7 1 7 0 7     15251.4779   0.0002  0.003
  9:  8 1 8 1 9   7 1 7 1 8     15251.5341   0.0010  0.003
 10:  8 1 8 0 9   7 1 7 0 8     15251.5483  -0.0015  0.003
 11:  6 1 6 1 6   5 1 5 1 5     11471.4171  -0.0003  0.003
 12:  6 1 6 0 6   5 1 5 0 5     11471.4340   0.0006  0.003
 13:  6 1 6 1 7   5 1 5 1 6     11471.5120   0.0000  0.003
 14:  6 1 6 0 7   5 1 5 0 6     11471.5285   0.0005  0.003
 15:  8 1 8 0 8   7 0 7 0 7     15560.2362   0.0000  0.003
 16:  8 1 8 1 8   7 0 7 1 7     15560.2496  -0.0021  0.003
 17:  9 1 9 0 9   8 0 8 0 8     17345.8797  -0.0020  0.003
 18:  9 1 9 1 9   8 0 8 1 8     17345.8953   0.0038  0.003
 19:  9 1 9 1 9   8 1 8 1 8     17133.5382   0.0021  0.003
 20:  9 1 9 0 9   8 1 8 0 8     17133.5558   0.0028  0.003
 21:  9 1 9 1 10   8 1 8 1 9     17133.6033   0.0023  0.003
 22:  9 1 9 0 10   8 1 8 0 9     17133.6192   0.0013  0.003
 23:  9 1 9 1 8   8 1 8 1 7     17133.5708  -0.0066  0.003
 24:  9 1 9 0 8   8 1 8 0 7     17133.5901  -0.0042  0.003
 25:  9 0 9 1 9   8 0 8 1 8     17204.1923   0.0001  0.003
 26:  9 0 9 0 9   8 0 8 0 8     17204.2035   0.0006  0.003
 27:  9 0 9 1 8   8 0 8 1 7     17204.2908  -0.0012  0.003
 28:  9 0 9 0 8   8 0 8 0 7     17204.3017  -0.0011  0.003
 29:  9 0 9 1 10   8 0 8 1 9     17204.3094   0.0015  0.003
 30:  9 0 9 0 10   8 0 8 0 9     17204.3204   0.0017  0.003
 31:  8 0 8 1 8   7 0 7 1 7     15347.8960  -0.0004  0.003
 32:  8 0 8 0 8   7 0 7 0 7     15347.9083   0.0009  0.003
 33:  8 0 8 1 7   7 0 7 1 6     15348.0184  -0.0018  0.003
 34:  8 0 8 0 7   7 0 7 0 6     15348.0295  -0.0017  0.003
 35:  8 0 8 1 9   7 0 7 1 8     15348.0382   0.0011  0.003
 36:  8 0 8 0 9   7 0 7 0 8     15348.0495   0.0013  0.003
 37:  7 0 7 1 7   6 0 6 1 6     13488.1131  -0.0007  0.003
 38:  7 0 7 0 7   6 0 6 0 6     13488.1259  -0.0004  0.003
```

39:	7 0 7 1 6	6 0 6 1 5	13488.2609	0.0021	0.003
40:	7 0 7 0 6	6 0 6 0 5	13488.2692	-0.0020	0.003
41:	7 0 7 1 8	6 0 6 1 7	13488.2794	0.0018	0.003
42:	7 0 7 0 8	6 0 6 0 7	13488.2905	0.0005	0.003
43:	6 0 6 1 6	5 0 5 1 5	11618.5842	-0.0005	0.003
44:	6 0 6 0 6	5 0 5 0 5	11618.5995	-0.0002	0.003
45:	6 0 6 1 5	5 0 5 1 4	11618.7397	0.0007	0.003
46:	6 0 6 0 5	5 0 5 0 4	11618.7520	-0.0019	0.003
47:	6 0 6 1 7	5 0 5 1 6	11618.7646	0.0017	0.003
48:	6 0 6 0 7	5 0 5 0 6	11618.7785	0.0006	0.003
49:	8 0 8 1 7	7 1 7 1 6	15038.9894	0.0034	0.003
50:	8 0 8 1 9	7 1 7 1 8	15039.0251	-0.0083	0.003
51:	8 0 8 0 7	7 1 7 0 6	15039.0372	0.0077	0.003
52:	8 0 8 0 9	7 1 7 0 8	15039.0731	-0.0037	0.003
53:	8 0 8 1 8	7 1 7 1 7	15039.1084	0.0029	0.003
54:	8 0 8 0 8	7 1 7 0 7	15039.1455	-0.0034	0.003
55:	7 1 7 0 7	6 0 6 0 6	13796.8866	0.0018	0.003
56:	7 1 7 1 7	6 0 6 1 6	13796.9021	-0.0026	0.003

PARAMETERS IN FIT (values truncated and Nlines statistics):

10000	A /MHz	2170.2055(79)	1
10011	A /MHz	2170.2155(79)	2
20000	B /MHz	1039.72015(93)	3
20011	B /MHz	1039.71300(93)	4
30000	C /MHz	933.51362(21)	5
30011	C /MHz	933.51363(21)	6
200	Del_J /kHz	[0.057601793]	7
211	Del_J /kHz	[0.052524468]	8
1100	Del_JK /kHz	[0.20474369]	9
1111	Del_JK /kHz	[0.22189703]	10
2000	Del_K /kHz	[-0.362910405]	11
2011	Del_K /kHz	[-0.457394903]	12

40100	del_J /kHz	[0.007341759839]	13
40111	del_J /kHz	[0.004716238693]	14
41000	del_K /kHz	[-0.474749131]	15
41011	del_K /kHz	[-0.563075176]	16
110010000	Xaa /MHz	-5.051(31)	17
-110010011	Xaa /MHz	-5.051(31)	= 1.00000 * 17
-110020000	Xaa /MHz	-5.051(31)	= 1.00000 * 17
-110020011	Xaa /MHz	-5.051(31)	= 1.00000 * 17
110030000	Xcc /MHz	2.08(16)	18
-110030011	Xcc /MHz	2.08(16)	= 1.00000 * 18
-110020000	Xcc /MHz	2.08(16)	= 1.00000 * 18
-110020011	Xcc /MHz	2.08(16)	= 1.00000 * 18

MICROWAVE AVG = 0.000005 MHz, IR AVG = 0.00000
MICROWAVE RMS = 0.002464 MHz, IR RMS = 0.00000
END OF ITERATION 1 OLD, NEW RMS ERROR= 0.82133 0.82133

distinct frequency lines in fit: 56
distinct parameters of fit: 8

MICROWAVE	lines fitted total	dv=0	dv.ne.0	lines UNFITTD	lines e>900	RMS	RMS ERROR	J range		Ka range		freq. range	
v"= 0	28	28	0	0	0	0.002249	0.74966	5	9	0	1	11471	17346
v"= 1	28	28	0	0	0	0.002601	0.86710	5	9	0	1	11471	17346
total:	56	56	0	0	0	0.002432	0.81051						

NOTE: the RMS values above are for Nlines statistics, but the 'total' values may differ slightly from those in the .FIT file since the o-c values for this evaluation are as rounded in the .FIT.

PARAMETERS IN FIT WITH STANDARD ERRORS ON THOSE THAT ARE FITTED:
(values rounded and degrees of freedom, Ndegf=Nlines-Nconst, statistics)

10000	A /MHz	2170.2055(70)	1
10011	A /MHz	2170.2155(70)	2
20000	B /MHz	1039.72015(83)	3
20011	B /MHz	1039.71300(83)	4

30000	C /MHz	933.51362(18)	5
30011	C /MHz	933.51364(18)	6
200	Del_J /kHz	[0.057601793]	7
211	Del_J /kHz	[0.052524468]	8
1100	Del_JK /kHz	[0.20474369]	9
1111	Del_JK /kHz	[0.22189703]	10
2000	Del_K /kHz	[-0.362910405]	11
2011	Del_K /kHz	[-0.457394903]	12
40100	del_J /kHz	[0.007341759839]	13
40111	del_J /kHz	[0.004716238693]	14
41000	del_K /kHz	[-0.474749131]	15
41011	del_K /kHz	[-0.563075176]	16
110010000	Xaa /MHz	-5.052(28)	17
-110010011	Xaa /MHz	-5.052(28)	= 1.00000 * 17
-110020000	Xaa /MHz	-5.052(28)	= 1.00000 * 17
-110020011	Xaa /MHz	-5.052(28)	= 1.00000 * 17
110030000	Xcc /MHz	2.08(14)	18
-110030011	Xcc /MHz	2.08(14)	= 1.00000 * 18
-110020000	Xcc /MHz	2.08(14)	= 1.00000 * 18
-110020011	Xcc /MHz	2.08(14)	= 1.00000 * 18

CORRELATION COEFFICIENTS, C.ij:

	A	A	B	B	C	C	Xaa	Xcc
A	1.0000							
A	0.0129	1.0000						
B	0.9448	-0.0021	1.0000					
B	-0.0021	0.9448	0.0034	1.0000				
C	-0.9559	0.0025	-0.9704	-0.0022	1.0000			
C	0.0025	-0.9559	-0.0022	-0.9704	0.0016	1.0000		
Xaa	-0.1118	-0.1118	0.0077	0.0077	-0.0156	-0.0156	1.0000	
Xcc	0.0130	0.0130	0.0528	0.0528	-0.0303	-0.0303	-0.2952	1.0000

Mean value of |C.ij|, i.ne.j = 0.2333
Mean value of C.ij, i.ne.j = -0.0861

No correlations with absolute value greater than 0.9950

Worst fitted lines (obs-calc/error):

50: -2.8	51: 2.6	23: -2.2	24: -1.4
18: 1.3	52: -1.2	54: -1.1	49: 1.1
53: 1.0	20: 0.9	56: -0.9	21: 0.8
19: 0.7	39: 0.7	16: -0.7	17: -0.7
40: -0.7	46: -0.6	55: 0.6	41: 0.6
33: -0.6	30: 0.6	47: 0.6	34: -0.6
10: -0.5	29: 0.5	22: 0.4	36: 0.4
27: -0.4	28: -0.4	35: 0.4	9: 0.3
4: 0.3	32: 0.3	2: 0.2	45: 0.2
37: -0.2	48: 0.2	12: 0.2	7: 0.2
26: 0.2	14: 0.2	43: -0.2	42: 0.2
3: -0.1	38: -0.1	31: -0.1	11: -0.1
44: -0.1	5: -0.1		

50:	8 0 8 1 9	7 1 7 1 8	15039.0251	-0.0083	0.003
51:	8 0 8 0 7	7 1 7 0 6	15039.0372	0.0077	0.003
23:	9 1 9 1 8	8 1 8 1 7	17133.5708	-0.0066	0.003
24:	9 1 9 0 8	8 1 8 0 7	17133.5901	-0.0042	0.003
18:	9 1 9 1 9	8 0 8 1 8	17345.8953	0.0038	0.003
52:	8 0 8 0 9	7 1 7 0 8	15039.0731	-0.0037	0.003
54:	8 0 8 0 8	7 1 7 0 7	15039.1455	-0.0034	0.003
49:	8 0 8 1 7	7 1 7 1 6	15038.9894	0.0034	0.003
53:	8 0 8 1 8	7 1 7 1 7	15039.1084	0.0029	0.003
20:	9 1 9 0 9	8 1 8 0 8	17133.5558	0.0028	0.003

/ SPFIT output reformatted with PIFORM

Spectroscopic transitions and quantum number assignments for hexafluoroacetone imine C(2)-13.

/ instead of : below denotes (o-c)>3*err

			obs	o-c	error o-c	blends wt	Notes
1:	9 1 9 1 9	8 1 8 1 8	17132.7238	0.0020	0.003		
2:	9 1 9 0 9	8 1 8 0 8	17132.7405	0.0025	0.003		
3:	9 1 9 1 8	8 1 8 1 7	17132.7568	-0.0067	0.003		
4/	9 1 9 0 8	8 1 8 1 7	17132.7753	-0.0115	0.003		
5:	9 1 9 1 10	8 1 8 1 9	17132.7886	0.0011	0.003		
6:	9 1 9 0 10	8 1 8 0 9	17132.8045	0.0008	0.003		
7:	7 1 7 1 7	6 1 6 1 6	13363.6864	-0.0009	0.003		
8:	7 1 7 0 7	6 1 6 0 6	13363.7027	0.0024	0.003		
9:	7 1 7 1 6	6 1 6 1 5	13363.7291	-0.0019	0.003		
10:	7 1 7 0 6	6 1 6 0 5	13363.7468	0.0027	0.003		
11:	7 1 7 1 8	6 1 6 1 7	13363.7667	-0.0029	0.003		
12:	7 1 7 0 8	6 1 6 0 7	13363.7835	0.0008	0.003		
13:	6 1 6 1 6	5 1 5 1 5	11470.8640	-0.0008	0.003		
14:	6 1 6 0 6	5 1 5 0 5	11470.8795	0.0032	0.003		
15:	6 1 6 1 7	5 1 5 1 6	11470.9582	-0.0026	0.003		
16:	6 1 6 0 7	5 1 5 0 6	11470.9744	0.0020	0.003		
17:	8 1 8 0 8	7 0 7 0 7	15559.6026	0.0014	0.003		
18:	8 1 8 1 8	7 0 7 1 7	15559.6182	-0.0028	0.003		
19:	9 1 9 0 9	8 0 8 0 8	17345.1425	-0.0034	0.003		
20:	9 1 9 1 9	8 0 8 1 8	17345.1571	0.0012	0.003		
21:	9 1 9 0 8	8 0 8 0 7	17345.3484	-0.0035	0.003		
22:	9 1 9 1 8	8 0 8 1 7	17345.3663	0.0044	0.003		
23/	9 1 9 1 10	8 0 8 1 9	17345.3793	0.0119	0.003		
24:	9 1 9 0 10	8 0 8 0 9	17345.3594	0.0020	0.003		
25:	8 1 8 1 9	7 1 7 1 8	15250.8060	-0.0004	0.003		
26:	8 1 8 0 9	7 1 7 0 8	15250.8198	-0.0011	0.003		
27:	5 0 5 1 5	4 0 4 1 4	9731.5983	-0.0065	0.003		
28:	5 0 5 0 5	4 0 4 0 4	9731.6169	0.0079	0.003		
29:	5 0 5 1 4	4 0 4 1 3	9731.7402	-0.0074	0.003		
30:	5 0 5 0 4	4 0 4 0 3	9731.7589	0.0071	0.003		
31:	5 0 5 1 6	4 0 4 1 5	9731.7779	-0.0072	0.003		
32:	5 0 5 0 6	4 0 4 0 5	9731.7957	0.0063	0.003		
33:	4 0 4 1 3	3 0 3 1 2	7823.0026	-0.0044	0.003		
34:	4 0 4 0 3	3 0 3 0 2	7823.0199	0.0082	0.003		
35:	4 0 4 1 5	3 0 3 1 4	7823.0713	-0.0064	0.003		
36:	4 0 4 0 5	3 0 3 0 4	7823.0889	0.0065	0.003		
37:	9 0 9 1 9	8 0 8 1 8	17203.3953	-0.0004	0.003		
38:	9 0 9 0 9	8 0 8 0 8	17203.4069	0.0021	0.003		

39:	9	0	9	1	8	8	0	8	1	7	17203.4937	-0.0031	0.003
40:	9	0	9	0	8	8	0	8	0	7	17203.5051	-0.0006	0.003
41:	9	0	9	1	10	8	0	8	1	9	17203.5125	-0.0003	0.003
42:	9	0	9	0	10	8	0	8	0	9	17203.5235	0.0016	0.003
43:	8	0	8	1	8	7	0	7	1	7	15347.1841	-0.0027	0.003
44:	8	0	8	0	8	7	0	7	0	7	15347.1962	0.0031	0.003
45:	8	0	8	1	7	7	0	7	1	6	15347.3070	-0.0050	0.003
46:	8	0	8	0	7	7	0	7	0	6	15347.3189	0.0007	0.003
47:	8	0	8	1	9	7	0	7	1	8	15347.3271	-0.0020	0.003
48:	8	0	8	0	9	7	0	7	0	8	15347.3386	0.0032	0.003
49:	7	0	7	1	7	6	0	6	1	6	13487.4834	-0.0047	0.003
50:	7	0	7	0	7	6	0	6	0	6	13487.4981	0.0055	0.003
51:	7	0	7	1	6	6	0	6	1	5	13487.6282	-0.0064	0.003
52:	7	0	7	0	6	6	0	6	0	5	13487.6412	0.0021	0.003
53:	7	0	7	1	8	6	0	6	1	7	13487.6497	-0.0040	0.003
54:	7	0	7	0	8	6	0	6	0	7	13487.6628	0.0046	0.003
55:	5	1	5	1	5	4	1	4	1	4	9571.8406	-0.0008	0.003
56:	5	1	5	0	5	4	1	4	0	4	9571.8495	-0.0018	0.003
57:	5	1	5	1	6	4	1	4	1	5	9571.9603	-0.0021	0.003
58:	5	1	5	0	6	4	1	4	0	5	9571.9731	0.0006	0.003
59:	6	0	6	1	6	5	0	5	1	5	11618.0354	-0.0050	0.003
60:	6	0	6	0	6	5	0	5	0	5	11618.0503	0.0060	0.003
61:	6	0	6	1	5	5	0	5	1	4	11618.1902	-0.0059	0.003
62:	6	0	6	0	5	5	0	5	0	4	11618.2035	0.0033	0.003
63:	6	0	6	1	7	5	0	5	1	6	11618.2159	-0.0047	0.003
64:	6	0	6	0	7	5	0	5	0	6	11618.2297	0.0052	0.003
65:	8	1	8	1	8	7	1	7	1	7	15250.7335	0.0001	0.003
66:	8	1	8	0	8	7	1	7	0	7	15250.7497	0.0019	0.003
67:	8	1	8	1	7	7	1	7	1	6	15250.7767	0.0000	0.003
68:	8	1	8	0	7	7	1	7	0	6	15250.7894	-0.0016	0.003
69:	8	1	8	1	9	7	1	7	1	8	15250.8060	-0.0004	0.003
70:	8	1	8	0	9	7	1	7	0	8	15250.8198	-0.0011	0.003
71:	8	0	8	1	8	7	1	7	1	7	15038.3010	0.0019	0.003
72:	8	0	8	0	8	7	1	7	0	7	15038.3391	-0.0006	0.003
73:	8	0	8	1	9	7	1	7	1	8	15038.2304	0.0039	0.003
74:	8	0	8	0	9	7	1	7	0	8	15038.2670	-0.0001	0.003
75:	8	0	8	1	7	7	1	7	1	6	15038.1826	0.0043	0.003
76:	8	0	8	0	7	7	1	7	0	6	15038.2186	-0.0004	0.003
77:	7	1	7	0	7	6	0	6	0	6	13796.3534	0.0074	0.003
78:	7	1	7	1	7	6	0	6	1	6	13796.3681	-0.0077	0.003

PARAMETERS IN FIT (values truncated and Nlines statistics):

10000	A /MHz	2170.1985(44)	1

10011	A /MHz	2170.2607(41)	2
20000	B /MHz	1039.65915(46)	3
20011	B /MHz	1039.65821(44)	4
30000	C /MHz	933.47028(12)	5
30011	C /MHz	933.46922(11)	6
200	Del_J /kHz	[0.057601793]	7
211	Del_J /kHz	[0.052524468]	8
1100	Del_JK /kHz	[0.20474369]	9
1111	Del_JK /kHz	[0.22189703]	10
2000	Del_K /kHz	[-0.362910405]	11
2011	Del_K /kHz	[-0.457394903]	12
40100	del_J /kHz	[0.007341759839]	13
40111	del_J /kHz	[0.004716238693]	14
41000	del_K /kHz	[-0.474749131]	15
41011	del_K /kHz	[-0.563075176]	16
110010000	Xaa /MHz	-5.108(24)	17
-110010011	Xaa /MHz	-5.108(24)	= 1.00000 * 17
-110020000	Xaa /MHz	-5.108(24)	= 1.00000 * 17
-110020011	Xaa /MHz	-5.108(24)	= 1.00000 * 17
110030000	Xcc /MHz	2.135(86)	18
-110030011	Xcc /MHz	2.135(86)	= 1.00000 * 18
-110020000	Xcc /MHz	2.135(86)	= 1.00000 * 18
-110020011	Xcc /MHz	2.135(86)	= 1.00000 * 18

MICROWAVE AVG = 0.000002 MHz, IR AVG = 0.00000
MICROWAVE RMS = 0.004334 MHz, IR RMS = 0.00000
END OF ITERATION 1 OLD, NEW RMS ERROR= 1.44480 1.44480

distinct frequency lines in fit: 78
distinct parameters of fit: 8

MICROWAVE	lines fitted total	dv=0	dv.ne.0	lines UNFITTD	lines e>900	RMS	RMS ERROR	J range		Ka range		freq. range	
v"= 0	38	38	0	0	0	0.003810	1.26986	3	9	0	1	7823	17345
v"= 1	40	39	1	0	0	0.004720	1.57328	3	9	0	1	7823	17345
total:	78	77	1	0	0	0.004301	1.43351						

NOTE: the RMS values above are for Nlines statistics, but the 'total' values may differ slightly from those in the .FIT file since the o-c values for this evaluation are as rounded in the .FIT.

PARAMETERS IN FIT WITH STANDARD ERRORS ON THOSE THAT ARE FITTED:
(values rounded and degrees of freedom, Ndegf=Nlines-Nconst, statistics)

10000	A /MHz	2170.1985(67)	1
10011	A /MHz	2170.2607(63)	2
20000	B /MHz	1039.65915(70)	3
20011	B /MHz	1039.65821(67)	4
30000	C /MHz	933.47028(18)	5
30011	C /MHz	933.46923(17)	6
200	Del_J /kHz	[0.057601793]	7
211	Del_J /kHz	[0.052524468]	8
1100	Del_JK /kHz	[0.20474369]	9
1111	Del_JK /kHz	[0.22189703]	10
2000	Del_K /kHz	[-0.362910405]	11
2011	Del_K /kHz	[-0.457394903]	12
40100	del_J /kHz	[0.007341759839]	13
40111	del_J /kHz	[0.004716238693]	14
41000	del_K /kHz	[-0.474749131]	15
41011	del_K /kHz	[-0.563075176]	16

110010000	Xaa /MHz	-5.109(37)	17
-110010011	Xaa /MHz	-5.109(37)	= 1.00000 * 17
-110020000	Xaa /MHz	-5.109(37)	= 1.00000 * 17
-110020011	Xaa /MHz	-5.109(37)	= 1.00000 * 17
110030000	Xcc /MHz	2.14(13)	18
-110030011	Xcc /MHz	2.14(13)	= 1.00000 * 18
-110020000	Xcc /MHz	2.14(13)	= 1.00000 * 18
-110020011	Xcc /MHz	2.14(13)	= 1.00000 * 18

CORRELATION COEFFICIENTS, C.ij:

	A	A	B	B	C	C	Xaa	Xcc
A	1.0000							
A	0.4969	1.0000						
B	0.8587	0.5102	1.0000					
B	0.5011	0.8416	0.5376	1.0000				
C	-0.9203	-0.3815	-0.9229	-0.3988	1.0000			
C	-0.4016	-0.9083	-0.4270	-0.9116	0.3172	1.0000		
Xaa	-0.1664	-0.1810	-0.0444	-0.0510	0.0507	0.0582	1.0000	
Xcc	0.1360	0.1205	0.1879	0.1746	-0.1346	-0.1218	-0.1963	1.0000

Mean value of |C.ij|, i.ne.j = 0.3914
Mean value of C.ij, i.ne.j = -0.0491

No correlations with absolute value greater than 0.9950

Worst fitted lines (obs-calc/error):

23: 4.0	4: -3.8	34: 2.7	28: 2.6
78: -2.6	77: 2.5	29: -2.5	31: -2.4
30: 2.4	3: -2.2	36: 2.2	27: -2.2
35: -2.1	51: -2.1	32: 2.1	60: 2.0
61: -2.0	50: 1.8	64: 1.7	59: -1.7
45: -1.7	63: -1.6	49: -1.6	54: 1.5
33: -1.5	22: 1.5	75: 1.4	53: -1.3
73: 1.3	21: -1.2	19: -1.1	62: 1.1
14: 1.1	48: 1.1	39: -1.0	44: 1.0
11: -1.0	18: -0.9	10: 0.9	43: -0.9
15: -0.9	2: 0.8	8: 0.8	52: 0.7
38: 0.7	57: -0.7	16: 0.7	47: -0.7
1: 0.7	24: 0.7		

23/ 9 1 9 1 10 8 0 8 1 9 17345.3793 0.0119 0.003

4/	9 1 9 0 8	8 1 8 1 7	17132.7753	-0.0115	0.003
34:	4 0 4 0 3	3 0 3 0 2	7823.0199	0.0082	0.003
28:	5 0 5 0 5	4 0 4 0 4	9731.6169	0.0079	0.003
78:	7 1 7 1 7	6 0 6 1 6	13796.3681	-0.0077	0.003
77:	7 1 7 0 7	6 0 6 0 6	13796.3534	0.0074	0.003
29:	5 0 5 1 4	4 0 4 1 3	9731.7402	-0.0074	0.003
31:	5 0 5 1 6	4 0 4 1 5	9731.7779	-0.0072	0.003
30:	5 0 5 0 4	4 0 4 0 3	9731.7589	0.0071	0.003
3:	9 1 9 1 8	8 1 8 1 7	17132.7568	-0.0067	0.003

/ SPFIT output reformatted with PIFORM

Spectroscopic transitions and quantum number assignments for hexafluoroacetone imine C(3)-13.

obs o-c error blends Notes
o-c wt
/ instead of : below denotes (o-c)>3*err

			obs	o-c	error
1:	5 1 5 1 5	4 1 4 1 4	9595.4892	0.0007	0.003
2:	5 1 5 0 5	4 1 4 0 4	9595.5018	0.0001	0.003
3:	5 1 5 1 4	4 1 4 1 3	9595.5378	0.0012	0.003
4:	5 1 5 0 4	4 1 4 0 3	9595.5499	0.0000	0.003
5:	5 1 5 1 6	4 1 4 1 5	9595.6092	0.0000	0.003
6:	5 1 5 0 6	4 1 4 0 5	9595.6219	-0.0005	0.003
7:	5 0 5 1 5	4 0 4 1 4	9755.6271	-0.0005	0.003
8:	5 0 5 0 5	4 0 4 0 4	9755.6451	-0.0003	0.003
9:	5 0 5 1 4	4 0 4 1 3	9755.7724	0.0005	0.003
10:	5 0 5 0 4	4 0 4 0 3	9755.7900	0.0003	0.003
11:	5 0 5 1 6	4 0 4 1 5	9755.8087	-0.0001	0.003
12:	5 0 5 0 6	4 0 4 0 5	9755.8263	-0.0004	0.003
13:	7 1 7 1 7	6 1 6 1 6	13395.6701	-0.0015	0.003
14:	7 1 7 0 7	6 1 6 0 6	13395.6863	0.0004	0.003
15:	7 1 7 1 6	6 1 6 1 5	13395.7169	0.0011	0.003
16:	7 1 7 0 6	6 1 6 0 5	13395.7308	0.0009	0.003
17:	7 1 7 1 8	6 1 6 1 7	13395.7524	-0.0015	0.003
18:	7 1 7 0 8	6 1 6 0 7	13395.7704	0.0023	0.003
19:	7 0 7 1 7	6 0 6 1 6	13517.9659	-0.0021	0.003
20:	7 0 7 0 7	6 0 6 0 6	13517.9805	0.0002	0.003
21:	7 0 7 1 6	6 0 6 1 5	13518.1147	0.0012	0.003
22:	7 0 7 0 6	6 0 6 0 5	13518.1236	-0.0020	0.003
23:	7 0 7 1 8	6 0 6 1 7	13518.1334	0.0007	0.003
24:	7 0 7 0 8	6 0 6 0 7	13518.1433	-0.0015	0.003
25:	4 0 4 1 4	3 0 3 1 3	7843.3258	0.0001	0.003
26:	4 0 4 0 4	3 0 3 0 3	7843.3418	-0.0028	0.003
27:	4 0 4 1 3	3 0 3 1 2	7843.4217	-0.0023	0.003
28:	4 0 4 0 3	3 0 3 0 2	7843.4417	-0.0013	0.003
29:	4 0 4 1 5	3 0 3 1 4	7843.4949	0.0012	0.003
30:	4 0 4 0 5	3 0 3 0 4	7843.5118	-0.0009	0.003
31:	5 1 5 1 5	4 0 4 1 4	10322.4212	-0.0051	0.003
32:	5 1 5 0 5	4 0 4 0 4	10322.4276	0.0072	0.003
33:	7 1 6 1 7	6 1 5 1 6	14111.7287	-0.0001	0.003
34:	7 1 6 0 7	6 1 5 0 6	14111.7831	-0.0010	0.003
35:	7 1 6 1 8	6 1 5 1 7	14111.8451	0.0001	0.003
36:	7 1 6 1 6	6 1 5 1 5	14111.8554	0.0014	0.003
37:	7 1 6 0 8	6 1 5 0 7	14111.8978	-0.0025	0.003
38:	7 1 6 0 6	6 1 5 0 5	14111.9111	0.0018	0.003

39:	4 1 3 1 4	3 1 2 1 3	8115.1188	0.0005 0.003
40:	4 1 3 0 4	3 1 2 0 3	8115.1601	0.0007 0.003
41:	4 1 3 1 5	3 1 2 1 4	8115.2631	0.0003 0.003
42:	4 1 3 0 5	3 1 2 0 4	8115.3043	0.0004 0.003
43:	8 0 8 1 7	7 1 7 1 6	15083.1101	-0.0006 0.003
44:	8 0 8 0 7	7 1 7 0 6	15083.1453	0.0011 0.003
45:	8 0 8 1 9	7 1 7 1 8	15083.1568	-0.0009 0.003
46:	8 0 8 0 9	7 1 7 0 8	15083.1918	0.0006 0.003
47:	8 0 8 1 8	7 1 7 1 7	15083.2232	-0.0003 0.003
48:	8 0 8 0 8	7 1 7 0 7	15083.2592	0.0022 0.003
49:	7 1 7 0 7	6 0 6 0 6	13815.9269	-0.0014 0.003
50:	7 1 7 1 7	6 0 6 1 6	13815.9403	0.0010 0.003
51:	7 1 7 0 8	6 0 6 0 7	13816.2952	-0.0041 0.003
52:	7 1 7 0 6	6 0 6 0 5	13816.3099	0.0001 0.003
53:	7 1 7 1 6	6 0 6 1 5	13816.3250	0.0041 0.003

PARAMETERS IN FIT (values truncated and Nlines statistics):

10000	A /MHz	2165.3027(24)	1
10011	A /MHz	2165.2925(25)	2
20000	B /MHz	1043.33694(20)	3
20011	B /MHz	1043.32924(20)	4
30000	C /MHz	935.530070(95)	5
30011	C /MHz	935.530474(96)	6
200	Del_J /kHz	[0.057601793]	7
211	Del_J /kHz	[0.052524468]	8
1100	Del_JK /kHz	[0.20474369]	9
1111	Del_JK /kHz	[0.22189703]	10
2000	Del_K /kHz	[-0.362910405]	11
2011	Del_K /kHz	[-0.457394903]	12
40100	del_J /kHz	[0.007341759839]	13
40111	del_J /kHz	[0.004716238693]	14

41000	del_K /kHz	[-0.474749131]	15
41011	del_K /kHz	[-0.563075176]	16
110010000	Xaa /MHz	-5.070(21)	17
-110010011	Xaa /MHz	-5.070(21)	= 1.00000 * 17
-110020000	Xaa /MHz	-5.070(21)	= 1.00000 * 17
-110020011	Xaa /MHz	-5.070(21)	= 1.00000 * 17
110030000	Xcc /MHz	2.109(67)	18
-110030011	Xcc /MHz	2.109(67)	= 1.00000 * 18
-110020000	Xcc /MHz	2.109(67)	= 1.00000 * 18
-110020011	Xcc /MHz	2.109(67)	= 1.00000 * 18

MICROWAVE AVG = -0.000021 MHz, IR AVG = 0.00000
MICROWAVE RMS = 0.001876 MHz, IR RMS = 0.00000
END OF ITERATION 1 OLD, NEW RMS ERROR= 0.62545 0.62545

distinct frequency lines in fit: 53
distinct parameters of fit: 8

MICROWAVE	lines fitted			lines	lines	RMS	RMS ERROR	J range		Ka range		freq. range	
	total	dv=0	dv.ne.0	UNFITTD	e>900								
v''= 0	27	27	0	0	0	0.002034	0.67787	3	8	0	1	7843	15083
v''= 1	26	26	0	0	0	0.001625	0.54156	3	8	0	1	7843	15083
total:	53	53	0	0	0	0.001844	0.61479						

NOTE: the RMS values above are for Nlines statistics, but the 'total' values may differ slightly from those in the .FIT file since the o-c values for this evaluation are as rounded in the .FIT.

PARAMETERS IN FIT WITH STANDARD ERRORS ON THOSE THAT ARE FITTED:
(values rounded and degrees of freedom, Ndegf=Nlines-Nconst, statistics)

10000	A /MHz	2165.3028(16)	1
10011	A /MHz	2165.2925(17)	2
20000	B /MHz	1043.33695(14)	3
20011	B /MHz	1043.32924(14)	4
30000	C /MHz	935.530070(64)	5
30011	C /MHz	935.530474(65)	6

200	Del_J /kHz	[0.057601793]	7
211	Del_J /kHz	[0.052524468]	8
1100	Del_JK /kHz	[0.20474369]	9
1111	Del_JK /kHz	[0.22189703]	10
2000	Del_K /kHz	[-0.362910405]	11
2011	Del_K /kHz	[-0.457394903]	12
40100	del_J /kHz	[0.007341759839]	13
40111	del_J /kHz	[0.004716238693]	14
41000	del_K /kHz	[-0.474749131]	15
41011	del_K /kHz	[-0.563075176]	16
110010000	Xaa /MHz	-5.070(15)	17
-110010011	Xaa /MHz	-5.070(15)	= 1.00000 * 17
-110020000	Xaa /MHz	-5.070(15)	= 1.00000 * 17
-110020011	Xaa /MHz	-5.070(15)	= 1.00000 * 17
110030000	Xcc /MHz	2.109(45)	18
-110030011	Xcc /MHz	2.109(45)	= 1.00000 * 18
-110020000	Xcc /MHz	2.109(45)	= 1.00000 * 18
-110020011	Xcc /MHz	2.109(45)	= 1.00000 * 18

CORRELATION COEFFICIENTS, C.ij:

	A	A	B	B	C	C	Xaa	Xcc
A	1.0000							
A	0.0669	1.0000						
B	0.5871	0.0180	1.0000					
B	0.0044	0.5305	0.0066	1.0000				
C	-0.7002	-0.0155	-0.6845	0.0002	1.0000			
C	-0.0006	-0.6227	0.0021	-0.6901	0.0006	1.0000		
Xaa	-0.2122	-0.2612	-0.0883	-0.0470	0.0424	-0.0100	1.0000	
Xcc	-0.0244	-0.0173	0.0722	0.0671	0.0227	0.0281	-0.3382	1.0000

Mean value of |C.ij|, i.ne.j = 0.1843
Mean value of C.ij, i.ne.j = -0.0808

No correlations with absolute value greater than 0.9950

Worst fitted lines (obs-calc/error):

32: 2.4	31: -1.7	53: 1.4	51: -1.4
26: -0.9	37: -0.8	18: 0.8	27: -0.8
48: 0.7	19: -0.7	22: -0.7	38: 0.6
13: -0.5	17: -0.5	24: -0.5	49: -0.5
36: 0.5	28: -0.4	29: 0.4	21: 0.4
3: 0.4	15: 0.4	44: 0.4	34: -0.3
50: 0.3	45: -0.3	16: 0.3	30: -0.3
40: 0.2	23: 0.2	1: 0.2	46: 0.2
43: -0.2	39: 0.2	6: -0.2	7: -0.2
9: 0.2	12: -0.1	14: 0.1	42: 0.1
10: 0.1	47: -0.1	41: 0.1	8: -0.1
20: 0.1	52: 0.0	35: 0.0	33: 0.0
25: 0.0	11: 0.0		

32:	5 1 5 0 5	4 0 4 0 4	10322.4276	0.0072	0.003
31:	5 1 5 1 5	4 0 4 1 4	10322.4212	-0.0051	0.003
53:	7 1 7 1 6	6 0 6 1 5	13816.3250	0.0041	0.003
51:	7 1 7 0 8	6 0 6 0 7	13816.2952	-0.0041	0.003
26:	4 0 4 0 4	3 0 3 0 3	7843.3418	-0.0028	0.003
37:	7 1 6 0 8	6 1 5 0 7	14111.8978	-0.0025	0.003
18:	7 1 7 0 8	6 1 6 0 7	13395.7704	0.0023	0.003
27:	4 0 4 1 3	3 0 3 1 2	7843.4217	-0.0023	0.003
48:	8 0 8 0 8	7 1 7 0 7	15083.2592	0.0022	0.003
19:	7 0 7 1 7	6 0 6 1 6	13517.9659	-0.0021	0.003

/ SPFIT output reformatted with PIFORM

Spectroscopic transitions and quantum number assignments for hexafluoroacetone imine N-15.

```
-----------------------------------------------------------------------------------------=========
                                        obs        o-c    error    blends     Notes
                                                          o-c      wt
   / instead of : below denotes (o-c)>3*err
-----------------------------------------------------------------------------------------=========
  1:  4  0  4  1    3  0  3  1          7809.7595   0.0023  0.003
  2:  4  0  4  0    3  0  3  0          7809.7747  -0.0002  0.003
  3:  5  0  5  1    4  1  4  1          9025.9579   0.0010  0.003
  4:  5  0  5  0    4  1  4  1          9026.0322   0.0040  0.003
  5:  5  0  5  1    4  0  4  1          9708.0735  -0.0007  0.003
  6:  5  0  5  0    4  0  4  0          9708.0903   0.0000  0.003
  7:  5  2  3  1    4  2  2  1         10001.4362  -0.0025  0.003
  8:  5  2  3  0    4  2  2  0         10001.4877  -0.0014  0.003
  9:  5  1  4  1    4  1  3  1         10107.1813   0.0059  0.003
 10:  5  1  4  0    4  1  3  0         10107.2237   0.0015  0.003
 11:  6  0  6  1    5  1  5  1         11061.3538  -0.0034  0.003
 12:  6  0  6  0    5  1  5  0         11061.3973  -0.0012  0.003
 13:  6  1  6  1    5  1  5  1         11438.8069  -0.0013  0.003
 14:  6  1  6  0    5  1  5  0         11438.8205  -0.0013  0.003
 15:  6  0  6  1    5  0  5  1         11582.4771  -0.0010  0.003
 16:  6  0  6  0    5  0  5  0         11582.4897  -0.0014  0.003
 17:  6  1  6  0    5  0  5  0         11959.9127  -0.0017  0.003
 18:  6  1  6  1    5  0  5  1         11959.9297   0.0005  0.003
 19:  7  1  7  0    6  0  6  0         13701.0991   0.0002  0.003
 20:  7  1  7  1    6  0  6  1         13701.1120  -0.0008  0.003
 21:  8  1  8  1    7  1  7  1         15202.2660  -0.0013  0.003
 22:  8  1  8  0    7  1  7  0         15202.2819   0.0010  0.003
 23:  8  0  8  1    7  0  7  1         15289.5646  -0.0018  0.003
 24:  8  0  8  0    7  0  7  0         15289.5766   0.0014  0.003
 25:  8  1  8  0    7  0  7  0         15463.2158  -0.0010  0.003
 26:  8  1  8  1    7  0  7  1         15463.2288   0.0010  0.003
 27/  8  4  5  1    7  4  4  1         15839.7794   0.0100  0.003
 28:  8  4  5  0    7  4  4  0         15839.8344   0.0057  0.003
 29:  8  3  6  1    7  3  5  1         15840.7698  -0.0003  0.003
 30:  8  3  6  0    7  3  5  0         15840.8282   0.0002  0.003
 31:  8  4  4  1    7  4  3  1         15844.6279  -0.0046  0.003
 32:  8  4  4  0    7  4  3  0         15844.6856  -0.0070  0.003
 33:  8  3  5  1    7  3  4  1         15931.2147   0.0010  0.003
 34:  8  3  5  0    7  3  4  0         15931.2812  -0.0030  0.003
 35:  8  1  7  1    7  1  6  1         16020.8646  -0.0020  0.003
 36:  8  1  7  0    7  1  6  0         16020.9157  -0.0012  0.003
 37:  8  2  6  1    7  2  5  1         16169.6312  -0.0024  0.003
 38:  8  2  6  0    7  2  5  0         16169.7137  -0.0001  0.003
 39:  9  0  9  1    8  1  8  1         16963.4348  -0.0008  0.003
 40:  9  0  9  0    8  1  8  0         16963.4625  -0.0008  0.003
```

```
 41:  9  1  9  0    8  0  8  0           17249.2161  -0.0009  0.003
 42:  9  1  9  1    8  0  8  1           17249.2286   0.0051  0.003
 43:  9  4  6  1    8  4  5  1           17831.5262   0.0028  0.003
 44:  9  4  6  0    8  4  5  0           17831.5922   0.0030  0.003
 45:  9  4  5  1    8  4  4  1           17843.0097  -0.0013  0.003
 46:  9  4  5  0    8  4  4  0           17843.0780  -0.0007  0.003
--------------------------------------------------------------------------------
```

PARAMETERS IN FIT (values truncated and Nlines statistics):

10000	A /MHz	2134.1857(28)	1
10011	A /MHz	2134.1836(21)	2
20000	B /MHz	1043.25063(14)	3
20011	B /MHz	1043.24305(13)	4
30000	C /MHz	929.59787(10)	5
30011	C /MHz	929.598229(92)	6
200	Del_J /kHz	[0.057601793]	7
211	Del_J /kHz	[0.052524468]	8
1100	Del_JK /kHz	[0.20474369]	9
1111	Del_JK /kHz	[0.22189703]	10
2000	Del_K /kHz	[-0.362910405]	11
2011	Del_K /kHz	[-0.457394903]	12
40100	del_J /kHz	[0.007341759839]	13
40111	del_J /kHz	[0.004716238693]	14
41000	del_K /kHz	[-0.474749131]	15
41011	del_K /kHz	[-0.563075176]	16

```
MICROWAVE AVG =        0.000009 MHz, IR AVG =       0.00000
MICROWAVE RMS =        0.002871 MHz, IR RMS =       0.00000
END OF ITERATION  1 OLD, NEW RMS ERROR=        0.95708         0.95708
```

distinct frequency lines in fit: 46
distinct parameters of fit: 6

MICROWAVE	lines fitted			lines UNFITTD	lines e>900	RMS	RMS ERROR	J range		Ka range		freq. range	
	total	dv=0	dv.ne.0										
v"= 0	22	22	0	0	0	0.002327	0.77574	3	9	0	4	7810	17843
v"= 1	24	23	1	0	0	0.003247	1.08248	3	9	0	4	7810	17843
total:	46	45	1	0	0	0.002845	0.94824						

NOTE: the RMS values above are for Nlines statistics, but the 'total' values may differ slightly from those in the .FIT file since the o-c values for this evaluation are as rounded in the .FIT.

PARAMETERS IN FIT WITH STANDARD ERRORS ON THOSE THAT ARE FITTED:
(values rounded and degrees of freedom, Ndegf=Nlines-Nconst, statistics)

10000	A /MHz	2134.1858(29)	1
10011	A /MHz	2134.1836(21)	2
20000	B /MHz	1043.25063(14)	3
20011	B /MHz	1043.24306(13)	4
30000	C /MHz	929.59788(10)	5
30011	C /MHz	929.598229(94)	6
200	Del_J /kHz	[0.057601793]	7
211	Del_J /kHz	[0.052524468]	8
1100	Del_JK /kHz	[0.20474369]	9
1111	Del_JK /kHz	[0.22189703]	10
2000	Del_K /kHz	[-0.362910405]	11
2011	Del_K /kHz	[-0.457394903]	12
40100	del_J /kHz	[0.007341759839]	13
40111	del_J /kHz	[0.004716238693]	14
41000	del_K /kHz	[-0.474749131]	15

41011 del_K /kHz [-0.563075176] 16

CORRELATION COEFFICIENTS, C.ij:

	A	A	B	B	C	C
A	1.0000					
A	0.0044	1.0000				
B	0.6417	0.0542	1.0000			
B	0.0024	0.5764	0.0305	1.0000		
C	-0.7478	0.0782	-0.7272	0.0439	1.0000	
C	-0.0013	-0.6850	-0.0156	-0.6690	-0.0226	1.0000

Mean value of |C.ij|, i.ne.j = 0.2867
Mean value of C.ij, i.ne.j = -0.0958

No correlations with absolute value greater than 0.9950

Worst fitted lines (obs-calc/error):

27: 3.3	32: -2.3	9: 2.0	28: 1.9
42: 1.7	31: -1.5	4: 1.3	11: -1.1
44: 1.0	34: -1.0	43: 0.9	7: -0.8
37: -0.8	1: 0.8	35: -0.7	23: -0.6
17: -0.6	10: 0.5	24: 0.5	8: -0.5
16: -0.5	21: -0.4	45: -0.4	14: -0.4
13: -0.4	12: -0.4	36: -0.4	3: 0.3
15: -0.3	26: 0.3	25: -0.3	22: 0.3
33: 0.3	41: -0.3	20: -0.3	40: -0.3
39: -0.3	46: -0.2	5: -0.2	18: 0.2
29: -0.1	2: -0.1	30: 0.1	19: 0.1
38: 0.0	6: 0.0		

27/ 8 4 5 1	7 4 4 1	15839.7794	0.0100	0.003
32: 8 4 4 0	7 4 3 0	15844.6856	-0.0070	0.003
9: 5 1 4 1	4 1 3 1	10107.1813	0.0059	0.003
28: 8 4 5 0	7 4 4 0	15839.8344	0.0057	0.003
42: 9 1 9 1	8 0 8 1	17249.2286	0.0051	0.003
31: 8 4 4 1	7 4 3 1	15844.6279	-0.0046	0.003
4: 5 0 5 0	4 1 4 1	9026.0322	0.0040	0.003
11: 6 0 6 1	5 1 5 1	11061.3538	-0.0034	0.003
44: 9 4 6 0	8 4 5 0	17831.5922	0.0030	0.003
34: 8 3 5 0	7 3 4 0	15931.2812	-0.0030	0.003

/ SPFIT output reformatted with PIFORM

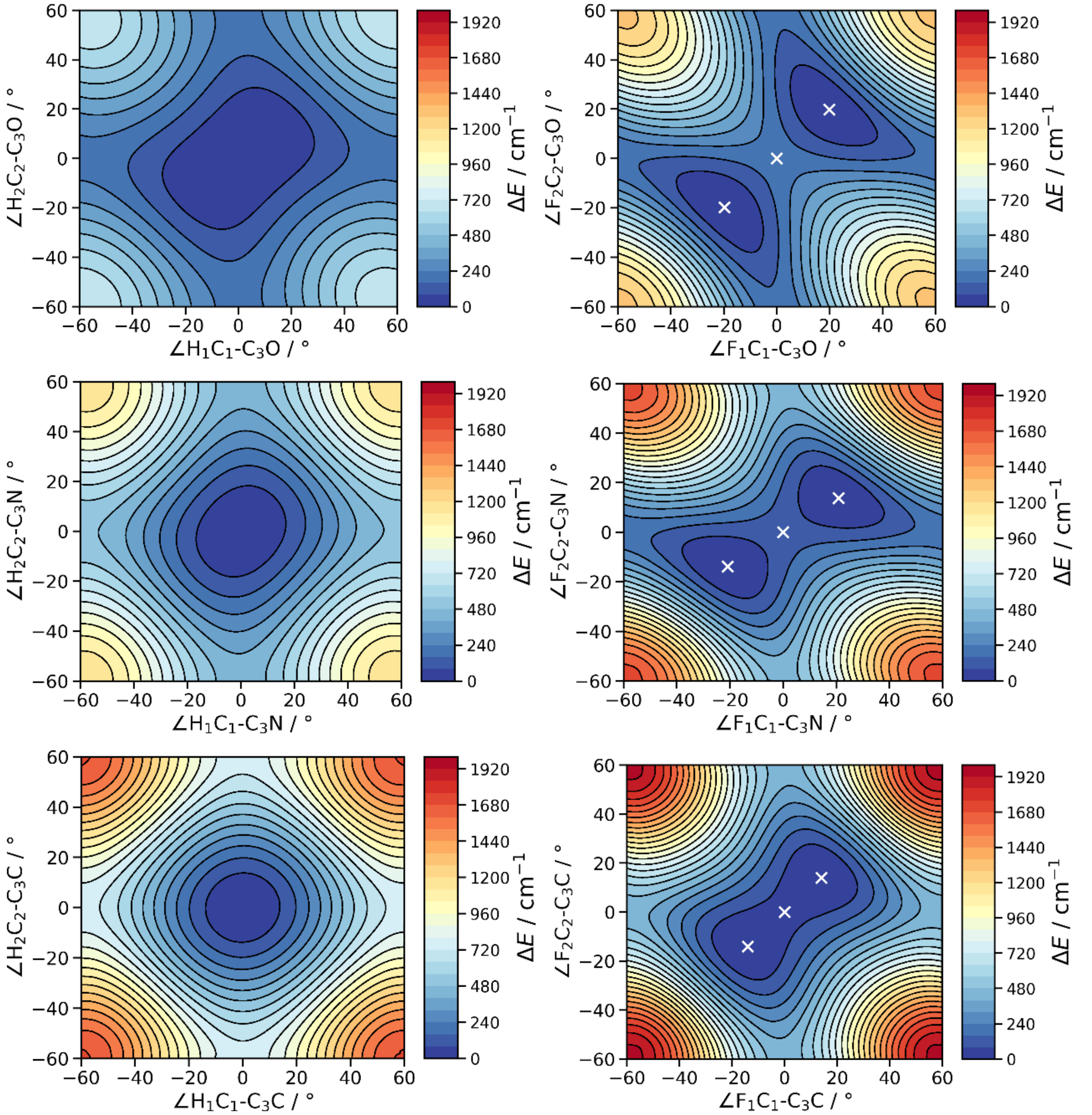

Supp Data Figure S1: 2-D potential energy scan for the six molecules of interest, acetone (top left), acetone imine (middle left), isobutene (bottom left), and, in each case, the hexafluorinated versions are shown to their right. Stationary points for the hexafluorinated species are shown with a white cross. See text for methods used.

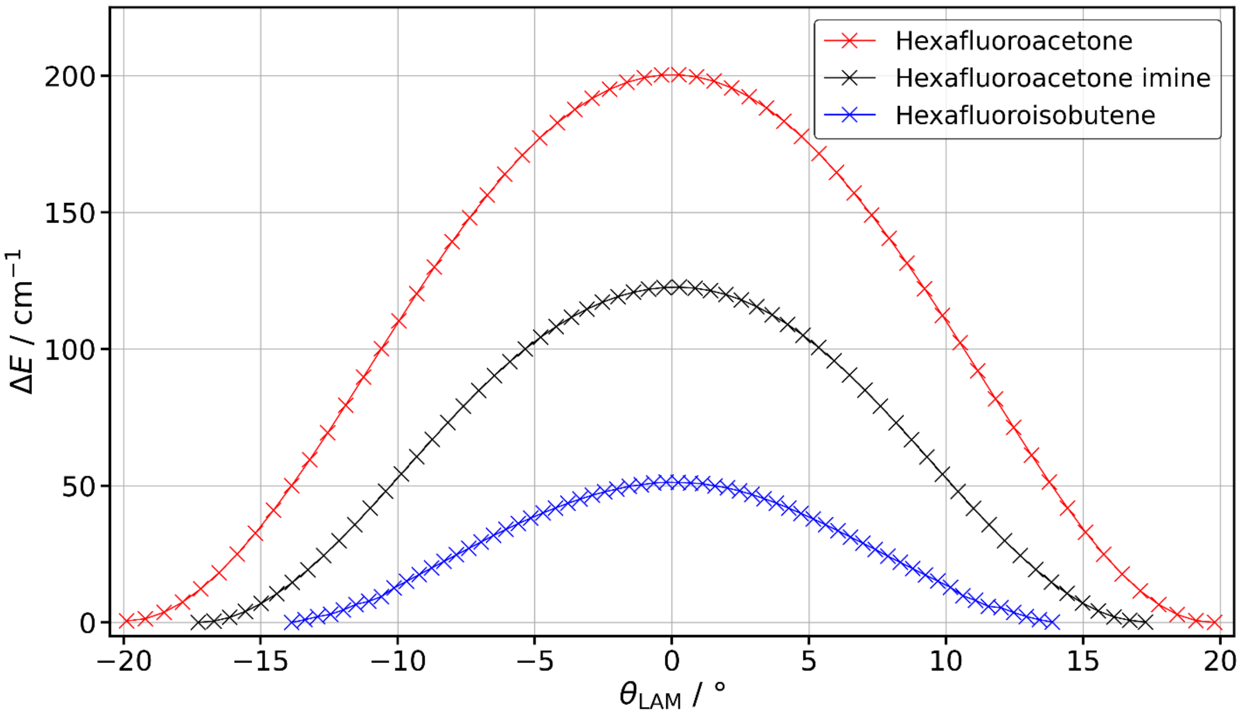

Supp Data Figure S2: NEB scans for the fluorinated variants using ORCA 6.0.1 and 60 images and B3LYP-D3(BJ)/aVTZ (using Gaussian's definition of B3LYP). The angle here is the average of the two dihedral angles defining the rotation of the CF3 groups.

Supp Data Table S2:

Overview of the electronic ($\Delta E^{\ddagger}_{\mathrm{el}}$) and zero-point corrected ($\Delta E^{\ddagger}_{0}$) barrier for the inversion. The singular imaginary mode ($\tilde{\nu}_{\mathrm{imag}}$) is also given associated with the transition state.

method	$\Delta E^{\ddagger}_{\mathrm{el}}$ / kJ mol^{-1}	$\Delta E^{\ddagger}_{0}$ / kJ mol^{-1}	$\tilde{\nu}_{\mathrm{imag}}$ / i cm^{-1}
B3LYP/6-311++G**	0.99	1.02	28.51
B3LYP-D3(BJ)/6-311++G**	1.07	1.10	29.43
B3LYP-D3(BJ)/aVTZ	1.41	1.39	31.99
PBE0-D3(BJ)/aVTZ	1.33	1.33	31.26
CAM-B3LYP-D3(BJ)/aVTZ	1.33	1.33	31.66
B2PLYP-D3(BJ)/aVTZ	1.51	1.50	33.00
DSD-PBEP86-D3(BJ)/aVTZ	1.65	1.64	34.15
MP2/6-311++G**	2.57	2.47	37.46
MP2/aVTZ	1.97	1.97	36.48
